# Pathobiological functions and clinical implications of annexin dysregulation in human cancers

**DOI:** 10.3389/fcell.2022.1009908

**Published:** 2022-09-28

**Authors:** Llara Prieto-Fernández, Sofía T. Menéndez, María Otero-Rosales, Irene Montoro-Jiménez, Francisco Hermida-Prado, Juana M. García-Pedrero, Saúl Álvarez-Teijeiro

**Affiliations:** ^1^ Department of Otolaryngology, Hospital Universitario Central de Asturias and Instituto de Investigación Sanitaria Del Principado de Asturias (ISPA), Instituto Universitario de Oncología Del Principado de Asturias (IUOPA), University of Oviedo, Oviedo, Spain; ^2^ CIBERONC, Instituto de Salud Carlos III, Madrid, Spain

**Keywords:** annexin, head and neck cancer, biomarker, diagnosis, therapeutic target

## Abstract

Annexins are an extensive superfamily of structurally related calcium- and phospholipid-binding proteins, largely conserved and widely distributed among species. Twelve human annexins have been identified, referred to as Annexin A1-13 (A12 remains as of yet unassigned), whose genes are spread throughout the genome on eight different chromosomes. According to their distinct tissue distribution and subcellular localization, annexins have been functionally implicated in a variety of biological processes relevant to both physiological and pathological conditions. Dysregulation of annexin expression patterns and functions has been revealed as a common feature in multiple cancers, thereby emerging as potential biomarkers and molecular targets for clinical application. Nevertheless, translation of this knowledge to the clinic requires in-depth functional and mechanistic characterization of dysregulated annexins for each individual cancer type, since each protein exhibits varying expression levels and phenotypic specificity depending on the tumor types. This review specifically and thoroughly examines the current knowledge on annexin dysfunctions in carcinogenesis. Hence, available data on expression levels, mechanism of action and pathophysiological effects of Annexin A1-13 among different cancers will be dissected, also further discussing future perspectives for potential applications as biomarkers for early diagnosis, prognosis and molecular-targeted therapies. Special attention is devoted to head and neck cancers (HNC), a complex and heterogeneous group of aggressive malignancies, often lately diagnosed, with high mortality, and scarce therapeutic options.

## Introduction

**FIGURE 1:**
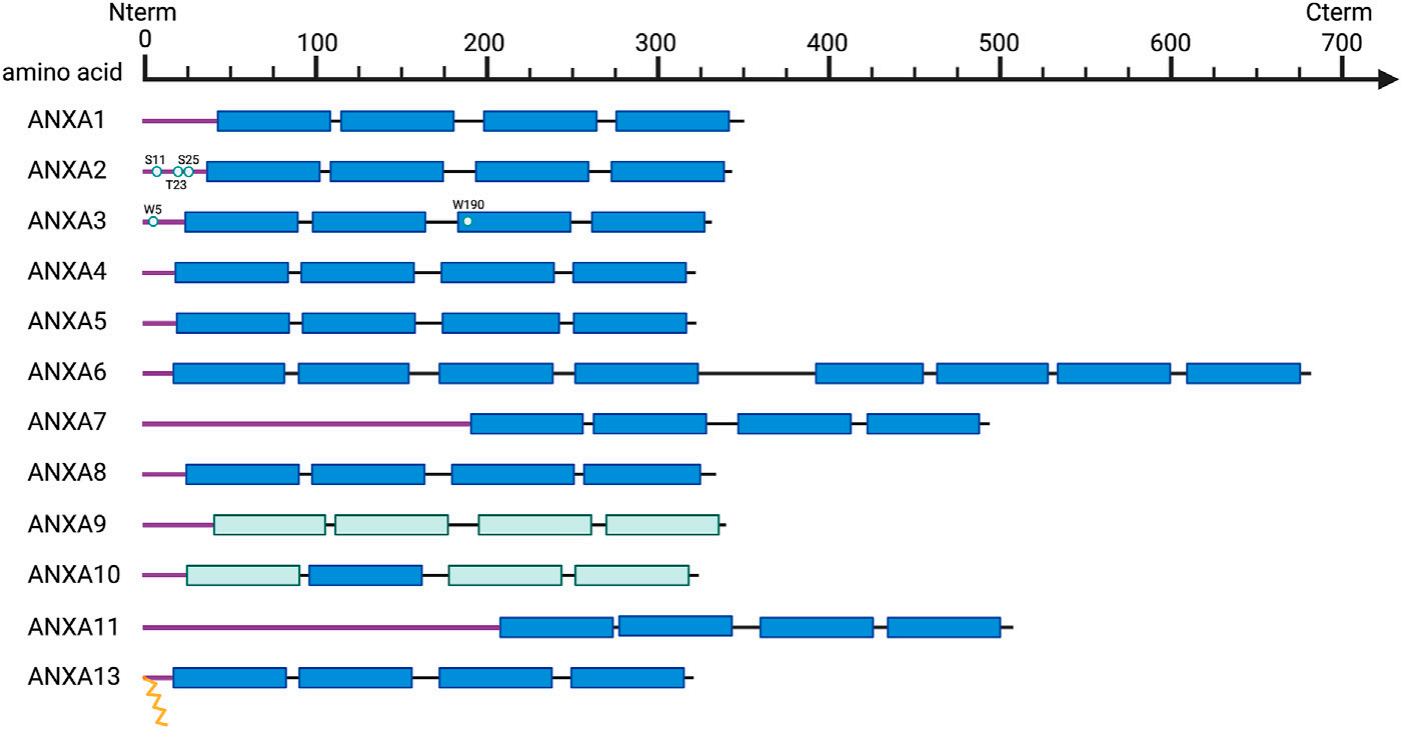
Schematic overview of annexin structural organization. Purple line, N-terminal tails; Dark blue, C-terminal core domains including four annexin repeats (duplicated to eight in ANXA6); Light blue, annexin repeats harboring non-functional type II calcium binding sites; Key residues are indicated and represented as spheres, and myristoylation as yellow zig-zag lines.

Annexins are an extensive multigene superfamily of proteins that possess high structural and biological homology (40–60%) (Gerke and Moss, 2002; Guo et al., 2013b) and whose main biochemical property is the binding or “annexing” to phospholipid membranes in a Ca2+-dependent manner (Moss and Morgan, 2004; Lim and Pervaiz, 2007). There are more than 500 annexins described in different species, which are widely distributed among eukaryotes, but largely absent in prokaryotes and yeasts (Fernandez and Morgan, 2003). The high evolutionary conservation of annexins among species and their presence in all higher eukaryotic organisms suggest an indispensable role in cell biology (Moss and Morgan, 2004). In humans, there are twelve annexins described (Figure 1), conventionally referred to as Annexin A1-13 (the ANXA12 gene is unassigned) (Morgan et al., 1999a), whose genes are spread throughout the genome on chromosomes 1, 2, 4, 5, 8, 9, 10 and 15 (Table 1) (Moss and Morgan, 2004).

**TABLE 1:** Overview of annexin expression dysregulation in human cancers vs. normal samples (unless stated otherwise). Transcriptomic and protein data was obtained from TCGA and CPTAC, respectively. TIMER 2.0 (http://timer.cistrome.org/) was used to asset differences in expression level whereas UALCAN webtool (http://ualcan.path.uab.edu/) was used for protein levels.

Overview of annexin expression dysregulation in human cancers VS. normal samples					
		mRNA		Protein	
Annexin	Locus	Upregulated	Downregulated	Upregulated	Downregulated
ANXA1	Chr9 q21.13	CESC, CHOL, GBM, KIRC, KIRP, LIHC, THCA	BRCA, HNSC, KICH, LUAD, PRAD, READ	KIRC, UCEC, PAAD, GBM	BRCA, COAD, LUAD, HNSC, LIHC
ANXA2	Chr15, q22.2	CESC, CHOL, COAD, ESCA, GBM, HNSC, KIRC, KIRP, LIHC, STAD, THCA, UCEC	BRCA, KICH, LUAD, PRAD	KIRC, PAAD, GBM, LIHC	BRCA, COAD, LUAD, HNSC
ANXA3	Chr 4, q21.21	CESC, CHOL, COAD, READ, SKMC (vs. metastasis), STAD, UCEC	BRCA, GBM, KICH, KIRC, LIHC, LUAD, LUSC, THCA	COAD, UCEC, PAAD	BRCA, KIRC, LUAD, GBM, LIHC
ANXA4	Chr 2, p13.3	BLCA, CHOL, COAD, ESCA, GBM, HNSC, KIRC, KIRP, LIHC, READ, STAD	KICH, LUAD, LUSC, PRAD, UCEC	COAD, KIRC, GBM	BRCA, OV, LUAD, PAAD, LIHC
ANXA5	Chr 4, q27	CHOL, COAD, ESCA, GBM, HNSC, KIRC, KIRP, LIHC, STAD, THCA	BLCA, BRCA, CESC, KICH, LUAD, LUSC, PCPG, UCEC	KIRC, PAAD, GBM	BRCA, OV, COAD, UCEC, LUAD, HNSC, LIHC
ANXA6	Chr 5, q33.1	KIRC, PCPG	BLCA, BRCA, CESC, GBM, KICH, KIRP, LUAD, LUSC, PRAD, SKCM (vs. metastasis), THCA, UCEC		BRCA, COAD, OV, KIRC, UCEC, LUAD, HNSC, GBM, LIHC
ANXA7	Chr 10, q22.2	CHOL, ESCA, HNSC, LIHC, LUAD, STAD	BLCA, COAD, GBM, KICH, KIRC, PRAD, READ, SKMC (vs. metastasis), THCA, UCEC	PAAD	BRCA, COAD, OV, KIRC, UCEC, LUAD, GBM, LIHC
ANXA8	Chr 10, q11.22	BLCA, CESC, COAD, ESCA, HNSC, KIRC, KIRP, LUSC, SKCM (vs. metastasis), STAD, THCA, UCEC	BRCA, GBM, KICH, LIHC, LUAD, PRAD		
ANXA9	Chr 1, q21.3	BRCA, CHOL, COAD, LIHC, LUAD, READ, SKCM (vs. metastasis), STAD	HNSC, KICH, KIRC, KIRP, LUSC, PRAD, THCA, UCEC		KIRC, LUAD, HNSC, PAAD, LIHC
ANXA10	Chr 4, q32.3	HNSC, LUAD, LUSC, PAAD, PRAD	CHOL, LIHC		
ANXA11	Chr 10, q22.3	CHOL, LIHC, THCA	BLCA, BRCA, COAD, GBM, HNSC, KICH, KIRC, LUSC, SKCM (vs. metastasis)	OV, PAAD	BRCA, COAD, KIRC, LUAD, HNSC, LIHC
ANXA13	Chr 8, q24.13	CHOL, ESCA, HNSC, KIRC, KIRP, LIHC, PAAD, STAD	BRCA, CESC, COAD, KICH, PRAD, UCEC	UCEC, HNSC	COAD, LIHC
		Data from TIMER 2.0 http://timer.cistrome.org		Data from UALCAN http://ualcan.path.uab.edu/analysis-prot.html	

BLCA, Bladder Urothelial Carcinoma; BRCA, Breast invasive carcinoma; CESC, Cervical squamous cell carcinoma and endocervical adenocarcinoma; CHOL, Cholangiocarcinoma; COAD, Colon adenocarcinoma; ESCA, Esophageal carcinoma; GBM, Glioblastoma multiforme; HNSC, Head and Neck squamous cell carcinoma (highlighted in bold); KICH, Kidney Chromophobe; KIRC, Kidney renal clear cell carcinoma; KIRP, Kidney renal papillary cell carcinoma; LIHC, Liver hepatocellular carcinoma; LUAD, Lung adenocarcinoma; LUSC, Lung squamous cell carcinoma; PAAD, Pancreatic adenocarcinoma; PCPG, Pheochromocytoma and Paraganglioma; PRAD, Prostate adenocarcinoma; OV, Ovarian serous cystadenocarcinoma; READ, Rectum adenocarcinoma; SKCM, Skin Cutaneous Melanoma; STAD, Stomach adenocarcinoma; UCEC, Uterine Corpus Endometrial Carcinoma.

Structurally, annexins are characterized by a highly conserved C-terminal core domain composed of at least four conserved structural repeats (each one of 70 amino acids long), where the calcium and phospholipids binding domains are located (Figure 1) (Mirsaeidi et al., 2016). In addition, each annexin has a unique N-terminal domain with a variable length and amino acid sequence, involved in protein-protein interactions and responsible for their biological and functional specificity (Figure 1) (Gerke and Moss, 2002; Mirsaeidi et al., 2016). This variable N-terminal region contains binding sites for multiple protein partners, including members of the calcium binding S100 family (Rescher and Gerke, 2008; Rintala-Dempsey et al., 2008), and also for various kinases related to signaling pathways, such as the proto-oncogene tyrosine-kinase Src and the calcium-controlled serine threonine kinase PKC (Kheifets et al., 2006; Hayes and Moss, 2009).

Annexins are usually cytosolic and soluble proteins with a stable form, but also detectable in the nucleus and the cell surface (Mirsaeidi et al., 2016). In response to specific stimuli, these proteins could be translocated through various types of intracellular membranes, and transported to the cell exterior via an endoplasmic reticulum/Golgi-dependent pathway (Boudhraa et al., 2016).

Regarding their functions, annexins have been implicated in a large variety of biological processes, and in the regulation of several cellular membrane functions, such as cell adhesion and morphology, vesicle organization, endo- and exocytosis, membrane trafficking and scaffolding, maintaining membrane stability under stress conditions or regulation of cytoskeleton dynamics (Hayes et al., 2004; Rescher and Gerke, 2004; Draeger et al., 2011; Schloer et al., 2018). Accordingly, these biochemical properties make annexins perfect candidates to transduce the extracellular stimulus across the membrane into the activation of intracellular signaling pathways to trigger multiple cellular responses such as proliferation, apoptosis, inflammatory activity, angiogenesis, immune response regulation, cell differentiation and also cell motility and invasion (Gerke and Moss, 2002; Ling et al., 2004; Schloer et al., 2018).

The expression levels and tissue distribution vary widely in both physiological and pathological conditions. Thus, some annexins such as Annexins A1, A2, A3, A4, A5, A6, A7 and A11 exhibit an ubiquitous expression, whereas others show very restrictive expression patterns, such as Annexin A8 in placenta and skin, Annexin A10 in stomach and Annexin A13 in small intestine (Fernandez and Morgan, 2003) (Table 2). Although direct evidence for a causative role of annexins in human diseases has not yet been demonstrated, several pathologies such as diabetes, cardiovascular and autoimmune diseases, infection and cancer have been associated with annexin dysfunctions, so termed “annexinopathies” (Rand, 1999; Hayes et al., 2007).

**TABLE 2:** Functional and mechanistic roles of annexin dysregulation in cancer biology.

Roles of annexin dysregulation in cancer biology				
Annexin	Tissue expression	Structural singularities	Biological function	Signaling pathways
ANXA1	Ubiquitous		Cell Proliferation; Apoptosis; Differentiation; Cell Migration; Invasion; Immunomodulation; Inflammation; Membrane remodeling; Membrane trafficking; Phagocytosis; Cell adhesion; Cell-cell communication; Autophagy (Alldridge and Bryant, 2003; Solito et al., 2003; Petrella et al., 2005; Lim and Pervaiz, 2007; D’Acquisto et al., 2008; Yi and Schnitzer, 2009; Li et al., 2011; Dalli et al., 2012; Yang et al., 2013; Cooray et al., 2013; Gastardelo et al., 2014; Bist et al., 2015; Zhu et al., 2018; Raulf et al., 2018)	GC mediator; EGFR, HGFR, PDGFR and PKC substrate; Regulation of NFκB, ERK-MAPK, Rho-GTPases, EGFR/STAT3, PI3K/AKT and TRAIL pathways; Participate in BAD dephosphorylation; FPR ligand; PLA2 inhibition; BECN1 inhibition; M2 macrophage differentiation (Davidson et al., 1991; Raynal and Pollard, 1994; Alldridge and Bryant, 2003; Solito et al., 2003; Petrella et al., 2005; Lim and Pervaiz, 2007; Zhang et al., 2010; Li et al., 2011; Cooray et al., 2013; Gastardelo et al., 2014; Bist et al., 2015; Gobbetti and Cooray, 2016; Zhu et al., 2018; Araújo et al., 2021)
ANXA2	Ubiquitous	Key phosphorylation residues Ser11, Ser25 and Tyr23 (Liu et al., 2003a)	Cell Proliferation; Differentiation; Cell Migration; Invasion; Membrane remodeling; Membrane trafficking; Immunomodulation; Angiogenesis (Creutz, 1992; Harder and Gerke, 1993; Merrifield et al., 2001; Zobiack et al., 2003; Benaud et al., 2004; Morel and Gruenberg, 2007; de Graauw et al., 2008; Bao et al., 2009; Shetty et al., 2012; Chao et al., 2015; Rocha et al., 2018; Mahdi et al., 2020; Mao et al., 2021)	Regulation of DOCK3/β-Cat/WAVE2, TGF-β, AKT, Twist/Snail and JNK/cJun pathways; Src/ANXA2/STAT3 and EphA2/YES1/ANXA2 axis (Rescher et al., 2008; Kanagasabai et al., 2010; Zheng et al., 2011; Wang et al., 2012; Chen et al., 2015; Cui et al., 2016; Feng et al., 2017; Rocha et al., 2018; Mao et al., 2021)
ANXA3	Ubiquitous	Two relevant tryptophan residues (W5 and W190) (Sopkova et al., 2009)	Cell Proliferation; Apoptosis; Cell Migration; Angiogenesis; Inflammation; Membrane remodeling; Membrane trafficking (Gerke and Moss, 2002; Park et al., 2005; Chong et al., 2010; Faugaret et al., 2011; Meng et al., 2019; Zhang et al., 2021b)	Regulation of ERK, JNK, PI3K/AKT and EGFR pathway; PLA2 inhibition (Coméra et al., 1990; Ruan et al., 2010; Tong et al., 2015; Xu et al., 2019a; Wan et al., 2020)
ANXA4	Ubiquitous		Cell Migration; Invasion; Membrane remodeling (Kaetzel et al., 2001; Jiang et al., 2018; Xu et al., 2019b; Wang et al., 2020; Florentsen et al., 2021)	Regulation of PI3K/AKT/eNOS pathway; Adenylyl cyclase 5 inhibition (Heinick et al., 2015, 2020; Xu et al., 2019b)
ANXA5	Ubiquitous		Cell Proliferation; Cell Migration; Invasion; Cell adhesion; Membrane remodeling (Bouter et al., 2015; Ding et al., 2017; Li et al., 2018; Sun et al., 2018; Bouvet et al., 2020)	Regulation of ERK pathway (Wang et al., 2021)
ANXA6	Ubiquitous	8 annexin repeats forming 2 cores in C-terminal (Qi et al., 2015)	Cell proliferation; Cell Migration; Invasion; Cell adhesion; Membrane remodeling; Membrane trafficking, Autophagy; Cholesterol homeostasis (Potez et al., 2011; Swaggart et al., 2014; Qi et al., 2015; Boye et al., 2017; Grewal et al., 2017)	Regulation of EGFR/Ras/MAPK, FAK/PI3K and YAP pathway; Regulation of calcium entry (Pons et al., 2001; Grewal et al., 2005, 2017; Monastyrskaya et al., 2009; Muga et al., 2009; Cornely et al., 2011; Koese et al., 2013; Wang et al., 2013; Qi et al., 2015)
ANXA7	Ubiquitous	Long hydrophobic N-terminal (Grewal et al., 2016)	Cell proliferation; Calcium homeostasis; Membrane trafficking; Aggregation of chromaffin granules (Gerke and Moss, 2002; Grewal et al., 2016)	Regulation of COX-dependent PGE2 production; Regulation of EGFR pathway; GTPase function (Grewal et al., 2016)
ANXA8	Placenta and skin		Cell Adhesion; Angiogenesis; Membrane remodeling; Membrane trafficking; Endosomes biology (Goebeler et al., 2006, 2008; Poeter et al., 2014; Heitzig et al., 2017)	Participation in VEGFR signaling (Heitzig et al., 2017)
ANXA9	Ubiquitous	Unable to bind calcium (Morgan et al., 1999a)	Cell proliferation; Cell Migration; Invasion; Epidermis biology (Boczonadi and Määttä, 2012; Yu et al., 2018; Zhou et al., 2021)	Regulation of TGF-β pathway (Zhou et al., 2021)
ANXA10	Stomach	Unable to bind calcium (Morgan et al., 1999b)	Transcription regulator (Quiskamp et al., 2014)	Regulation of Akt and ERK/MAPK pathway (Kodaira et al., 2019)
ANXA11	Ubiquitous	Long hidrofobic N-terminal (Gerke and Moss, 2002)	Cell proliferation; Apoptosis; Membrane remodeling; Membrane trafficking; Sex differentiation (Wang et al., 2014)	Regulation of Cell cycle progression (Wang et al., 2014)
ANXA13	Small intestine	Myristoylated at N-terminal (Gerke and Moss, 2002)	Membrane trafficking; Lipid-raft dynamics (Lafont et al., 1998; Plant et al., 2000)	

Growing evidences have revealed that annexins are frequently and commonly dysregulated in multiple cancers, including HNC. As summarized in Table 1, altered expression levels of each annexin (both mRNA and protein) are frequently detected either upregulated or downregulated depending on the cancer type. Not surprisingly, annexin expression changes cause widespread functional effects on multiple biological and cellular processes including various cancer hallmarks (Table 2). On this basis, several annexins have emerged as potential biomarkers for cancer diagnosis, prognosis, disease monitoring, prediction of treatment response and/or therapeutic targets (Table 3).

**TABLE 3:** Clinical significance and potential therapeutic implications of annexin dysregulation in human cancers.

Clinical and therapeutic relevance of annexins					
Annexin	Good prognosis	Poor prognosis	Diagnosis/Stratification	Therapy resistance	Potential targeted therapy
ANXA1	Low: renal cancer Fu et al. (2020)	High: HER2+ BC; ESCC Paweletz et al. (2000) Low: HNSCC Garcia Pedrero et al. (2004)	Low, HNSCC differentiation grading, detection of epithelial dysplasia Garcia Pedrero et al. (2004); low, OSCC blood biomarker Faria et al. (2010)	ESCC Han et al. (2018); multiple myeloma Jia et al. (2018); NPC Huang et al. (2016)	Ac 2-26 Cardin et al. (2019); Guan et al. (2019)
	High: OC Fu et al. (2020)				
ANXA2	High: osteosarcoma Gillette et al. (2004)	High: ESCC Ma et al. (2014); NPC Chen et al. (2015); NSLC; HCC; OC; BC Li et al. (2021b) Low: HNSCC Rodrigo et al., 2011b. (2014), sinonasal adenocarcinoma Rodrigo et al. (2011a)		NPC Chen et al. (2015); BC Madureira et al. (2012); Grindheim et al. (2016)	CLG-shANXA2 Andey et al. (2014); anti-ANXA2 antibodies Lokman et al. (2013); ANXA2-targeting peptide motif CBP12 Staquicini et al. (2017), Lm-ANXA2 Kim et al. (2019)
ANXA3		High: GC; HCC; BC Liu et al. (2021a)	Prostate cancer Schostak et al. (2009)	HCC; OC; lung cancer; prostate cancer; BC; CRC Liu et al. (2021a)	
ANXA4		High: OSCC Liu et al. (2016a); OC Choi et al. (2013)	OSCC lepcha et al. (2021)	MESO Yamashita et al. (2012); NSCLC Gaudio et al. (2016); Zheng et al. (2018); Scala et al. (2021); OSCC Xiong et al. (2011); OC Morimoto et al. (2014)	
ANXA5	Positive: adult AML Niu et al. (2019)	High: glioma Zhong et al. (2021); HCC Sun et al. (2018); bladder Wu et al. (2021); CRC Xue et al. (2009); CSCC Shapanis et al. (2021)		NPC (Tang et al., 2012)	
ANXA6		Low: CC Sun et al. (2020); basal-like BC* Koumangoye et al. (2013) High: PDA Leca et al. (2016)	CC (Lomnytska et al., 2010); esophageal adenocarcinoma (Zaidi et al., 2014); HCC (Meier et al., 2016); OC (Noreen et al., 2020)	TNBC Koumangoye et al. (2013); Widatalla et al. (2019); Li et al. (2021a)	
ANXA7	High: GBM Hung and Howng. (2003)	High: GC^a^ Yuan et al. (2014); HCC Sun et al. (2009a); HCC Sun et al. (2009b); HER2- BC Srivastava et al. (2001a), Srivastava et al. (2004). Low: prostate Srivastava et al. (2001a), Srivastava et al. (2004).	Melanoma less invasive subtypes Kataoka et al. (2000); HER2- BC Srivastava et al. (2004)		
ANXA8		High: GC Ma et al. (2020); pancreatic cancer Pimiento et al. (2015); OC Gou et al. (2019); Zhu et al. (2020); OSCC Oka et al. (2016)	ER- basal-like DCIS subgroup Rossetti et al. (2016)		
ANXA9		High: CRC Miyoshi et al. (2014); Yu et al. (2018); BC Xiao et al. (2019); GC Zhou et al. (2021); OC Kou et al. (2021)	HNSCC differentiation grading Salom et al. (2019)	OC Kou et al. (2021)	
ANXA10		High: LUAD Yumura et al. (2022); GBM Xu et al. (2021); OC Wang et al. (2019); PDAC Ishikawa et al. (2022b); small bowel adenocarcinoma Ishikawa et al. (2021); PTC Liu et al. (2021b); CRC Bae et al. (2015); ESCC Kodaira et al. (2019); intrahepatic cholangiocarcinoma Shao et al. (2022); melanoma Zhang et al. (2021a) Low: early GC Ishikawa et al. (2020); bladder Munksgaard et al. (2011); HCC Liu et al. (2002), HCC Liu et al. (2012)	Serrated neoplasia pathway in CRC Bae et al. (2015); specific biomarker for gastrointestinal and pancreatic adenocarcinomas Lu et al. (2013); PDA Zhu et al. (2017)		
ANXA11	High: bladder Yao et al. (2022)	High: GC Hua et al. (2018); CRC Duncan et al. (2008); AML Song et al. (2022)	Bladder cancer Wu et al. (2021)	OC Song et al. (2007); mCRC Kim et al. (2011), Kim et al. (2013); Roh et al. (2016); AML Song et al. (2022)	
ANXA13		High: CRC Jiang et al. (2017); LUAD Xue et al. (2020)			

a Controversial.

Red: tumor-promoter role.

Green: tumor-suppressor role.

AD, Adenocarcinoma; AML, Acute myeloid leukemia; BC, Breast carcinoma; CC, Cervical cancer; CRC, Colorectal carcinoma; mCRC, metastatic colorectal carcinoma; CSCC, cutaneous squamous cell carcinoma; DCIS, Ductal carcinoma in situ; ESCC, Esophageal squamous cell carcinoma; ER, Estrogen receptor; GBM, Glioblastoma multiforme; CBP12, Colorectal cancer binding peptide; GC, Gastric cancer; CLG, Cationic ligand-guide; HCC, hepatocellular carcinoma; HER2+/−, human epidermal growth factor receptor 2; HNSCC, Head and neck squamous cell carcinoma; LUAD, Lung adenocarcinoma; MESO, Mesothelioma; NPC, Nasopharyngeal cancer; NSCLC, Non-small cell lung cancer; PDA, Pancreatic ductal adenocarcinoma; PTC, Papillary thyroid cancer; OC, Ovarian carcinoma; OSCC, Oral squamous cell carcinoma; TNBC, Triple-negative breast cancer.

**FIGURE 2:**
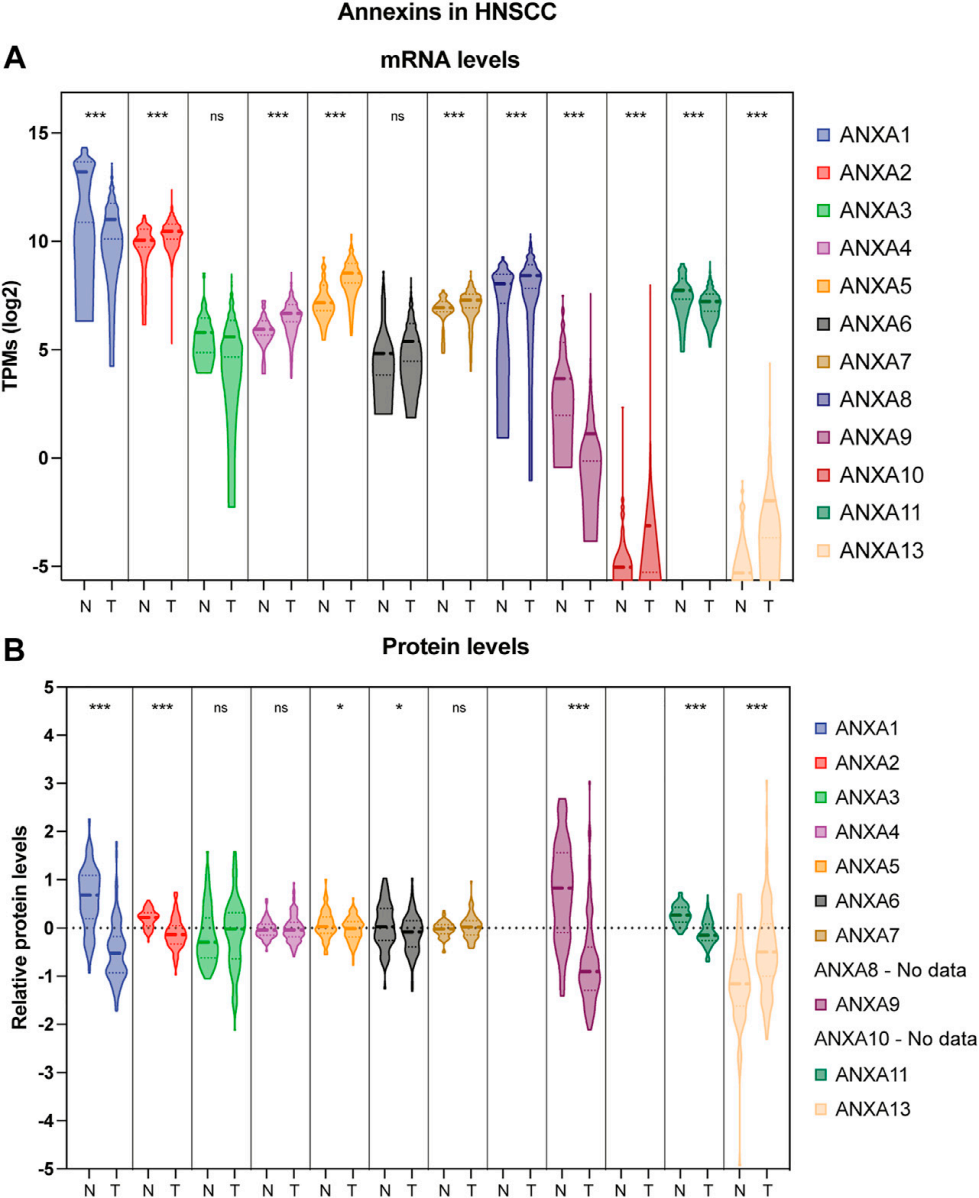
Violin plot comparison of annexin expression levels in HNSCC patient samples *versus* normal adjacent tissue. **(A)** Transcriptomic expression data were obtained at Xena repository (Goldman et al., 2020) from the TCGA-HNSCC cohort consisting of 44 normal adjacent tissue (N) and 522 primary tumors (T). Transcript per million (TPMs) are shown as log2 transformed (***p < 0.001 by *t*-test using Welch’s correction; ns, not significant). **(B)** Proteomic expression data from 72 normal adjacent tissue (N) and 110 primary tumors (T) were obtained from Proteomic Data Commons (https://pdc.cancer.gov/pdc/study/PDC000221). Ion intensity is shown as log2 transformed (***p < 0.001, *p < 0.05 by *t*-test using Welch’s correction; ns, not significant).

Head and neck cancers (HNC) represent the seventh most common cancer worldwide, comprising a highly diverse and heterogeneous group of malignancies (Mody et al., 2021). Most head and neck malignancies are diagnosed at a late stage due to the scarcity of specific symptoms. Mortality remains high mainly related to locoregional recurrences and second primary tumors (Leemans et al., 2011). Despite continuous advancements in the different treatment modalities (surgery, radio- and chemotherapy), survival rates for HNSCC patients have not been substantially improved in recent decades. At present, there are only few molecular-targeted therapies approved by the FDA: cetuximab (anti-EGFR) (Vermorken et al., 2007), nivolumab and pembrolizumab (anti-PD-1/PD-L1) (Cramer et al., 2019); however, these treatments only benefit 20–30% of patients (Cohen et al., 2019). Hence, there is an urgent need for novel therapeutic targets and more accurate prognosticators and early diagnosis markers that augment the limited predictability of the current clinicopathologic criteria. This review thoroughly and critically examines current knowledge and reported data on annexin dysregulation in human cancers with a special focus on HNC pathogenesis. An overview of all the annexins aberrantly expressed in HNC (either upregulated or downregulated) compared to the levels in normal tissue is depicted in Figure 2 (both mRNA and protein). In the following subsections, available data on expression dysregulation of Annexin A1-13 and the clinical and pathobiological relevance of each protein will be jointly reviewed, also further discussing future perspectives for their potential applications as biomarkers for early diagnosis, prognosis and molecular-targeted therapies.

## Annexin A1

### Structural and functional characteristics

Annexin A1 (ANXA1) was first described in the 1970s, characterized as the first member of the annexin superfamily, and called macrocortin, renocortin and lipomodulin, lipocortin-1 and lastly named Annexin A1. The *ANXA1* gene maps in the chromosomal region 9q12-q21.2 and encodes a 37-kDa protein, whose structural features are similar to those of the annexin superfamily members, and with a variable N-terminal regulatory region that contains sites of phosphorylation, glycosylation, acetylation and proteolysis, conferring as such specific biological properties (Buckingham et al., 2006; Guo et al., 2013b)**.** ANXA1 can be found in alternatively spliced isoforms, as proteolytic fragments of its bioactive N-terminus, interacting with different ligands, and hence could be localized in the nucleus, cytoplasm, membrane or extracellular matrix (Boudhraa et al., 2016). ANXA1 was first identified as an endogenous mediator of the anti-inflammatory effects of the glucocorticoids. Initially, ANXA1 was studied in neutrophils, eosinophils and monocytes where it is highly expressed (Perretti et al., 1996; Hannon et al., 2003; Perretti and Solito, 2004), and thereafter, it was widely detected in different tissues and involved in multiple cell processes, including cell survival, proliferation, apoptosis, differentiation and migration (Lim and Pervaiz, 2007).

ANXA1 is well-known by its participation in the inhibition of glucocorticoid-induced eicosanoid and phospholipase A2 (PLA2) synthesis (Raynal and Pollard, 1994; Lim and Pervaiz, 2007). ANXA1 is considered a mediator of the anti-inflammatory, immunosuppressive and antipyretic action of glucocorticoids, thereby regulating their expression and secretion (Davidson et al., 1991; Gobbetti and Cooray, 2016). It has also been reported that ANXA1 may act as an endogenous inhibitor of NFκB, inducible in response to anti-inflammatory agents (Zhang et al., 2010). Moreover, ANXA1 is a major substrate for numerous kinases, such as epidermal growth factor receptor (EGFR), hepatocyte growth factor receptor (HGFR/MET), platelet-derived growth factor receptor (PDGFR) and protein kinase C (PKC) (Lim and Pervaiz, 2007). These phosphorylation events lead to the activation of several signaling pathways (e.g., ERK-MAPK pathway), regulating cell proliferation and differentiation (Alldridge and Bryant, 2003). Seemingly, ANXA1 may create varying regulatory signals on different pathways that could explain its dual role, either promoting or inhibiting cell proliferation, as well as distinct functions and phenotypes depending on the cellular and tissue context.

ANXA1 may induce apoptosis through calcium release and BAD proteins dephosphorylation, allowing BAD association to mitochondria (Solito et al., 2003). During apoptosis, ANXA1 itself translocate to the nucleus, which can be inhibited by Bcl-2. ANXA1 can also regulate apoptosis through TRAIL (Petrella et al., 2005). By contrast, some groups have reported that ANXA1 exhibits anti-apoptotic properties, probably because its role depends on the cell type or cellular differentiation status. ANXA1 is also able to mediate the phagocytosis of apoptotic cells when it is recruited to the cell surface, where it binds to phosphatidylserine (PS) (Dalli et al., 2012).

ANXA1 has also been involved in motility and invasion processes, since mice with ANXA1 overexpression exhibited a significantly higher wound closure ability, and conversely, ANXA1 inhibition led to a reduction in the wound healing ability (Yi and Schnitzer, 2009). It has been described that the participation of ANXA1 in would healing is mediated by different signaling pathways such as MAPK, Rho-GTPases and NFκB (Bist et al., 2015).

Besides, special attention has been focused on studying the functional roles in membrane remodeling, cell adhesion, migration and cell signaling through the formyl peptide receptor (FPR), known receptors for externalized ANXA1 (Cooray et al., 2013; Gastardelo et al., 2014). FPRs may cause potent and opposite effects depending on the ligand (Cooray et al., 2013), and ANXA1, having the strongest affinity for FPR2, triggers different regulatory signaling pathways in a dose-dependent manner (Karlsson et al., 2005). This could plausibly explain how ANXA1 may elicit distinct regulatory signals and pleiotropic functions in different tissues (Boudhraa et al., 2016).

Noteworthy, during the last years, ANXA1 has emerged as an immunomodulatory protein and great effort has been devoted to understand its specific immune-suppressive role during malignant transformation. In physiological conditions, ANXA1 promotes immune suppression to counteract inflammatory process, specifically enhancing the differentiation of macrophages into M2 (Li et al., 2011). In cancer context, ANXA1 regulates macrophages activation by inhibiting the expression and activation of the inducible nitric oxide synthase (iNOS) (Smyth et al., 2006) or regulating nuclear EGFR and EGFR/STAT3 signaling pathway to ultimately create an immunosuppressive environment that facilitates cancer progression (Araújo et al., 2021).

### Altered expression and pathobiological role in cancer

ANXA1 dysregulation has been frequently detected in many types of cancer; however, its specific role has not yet been fully deciphered (Fu et al., 2020). ANXA1 has been found overexpressed in gastric cancer (Hippo et al., 2001), pancreatic and hepatocellular carcinoma (Masaki et al., 1996), colorectal cancer (Roth et al., 2010), lung cancer (Rong et al., 2014), melanoma (Rondepierre et al., 2009), skin cancer (Hummerich et al., 2006) and endometrial carcinoma (Voisin et al., 2011). On the other hand, ANXA1 is markedly down-regulated in breast cancer (Anbalagan et al., 2014; Yuan et al., 2016), prostate cancer (Kang et al., 2002), esophageal cancer (Paweletz et al., 2000), cervical cancer (Liu et al., 2011), lymphoma (Santos et al., 2009), hilar cholangiocarcinoma (Wang et al., 2010), intestinal-type sinonasal adenocarcinoma (Rodrigo et al., 2011a) and also in HNSCC (Garcia Pedrero et al., 2004).

The opposite expression levels of ANXA1 in different tumor types makes difficult to understand precisely the role played by ANXA1 during tumorigenesis. Actually, its action shows cellular or tissue specificity that could be due to post-translational modifications, potential site re-processing or epigenetic regulation among others. In fact, the contrasting patterns of ANXA1 expression in different tumor types is just one of the enigmas in deciphering the underlying regulatory mechanisms and phenotypic specificity of ANXA1.

### Therapeutic implications

ANXA1 has been related to treatment resistance in several cancers. It has been reported that serum ANXA1 levels increased after chemoradiotherapy in esophageal squamous cell carcinoma (ESCC) patients (Han et al., 2018). ANXA1 knockdown enhances the antitumor effect of bortezomib in multiple myeloma (Jia et al., 2018). In HNSCC and nasopharyngeal cancer (NPC), ANXA1 expression has been correlated with radiation resistance (Suh et al., 2015; Huang et al., 2016). These data underscore that ANXA1 could serve as a novel predictive biomarker of treatment response, and emerge as a potential co-adjuvant treatment to improve chemosensitivity in different types of cancer, including HNSCC.

It has been demonstrated that ANXA1 promotes the switching of macrophages to the protumoral M2 phenotype preventing the induction of cytotoxic T cell response, thus creating an immunosuppressed tumor microenvironment that facilitates tumor progression and dissemination (Araújo et al., 2021).

ANXA1 was the first annexin found to be implicated in the plasma membrane repair response. It is recruited within seconds at the wound region after an injury, demonstrating its function as stress-responsive protein (McNeil et al., 2006). In this context, cancer cells are normally under higher stress levels than normal cells, leading to plasma membrane damage, so ANXA1 could be a promising adjuvant treatment combined with current oncologic treatments to prevent plasma membrane repair in cancer cells after anti-proliferative treatments.

ANXA1 could be therapeutically exploited through its known receptors, FPR1 and FPR2. These receptors are predominantly expressed on the surface of several types of immune cells (macrophages, dendritic cells, neutrophils … ) as well as endothelial and epithelial cells (Rescher et al., 2002). Since ANXA1 expression is closely related to inflammatory processes, the signaling axis ANXA1/FPR could constitute an attractive immunomodulatory target for cancer therapies. ANXA1 regulates apoptosis and clearance of neutrophils and promotes monocytes recruitment during inflammatory events. However, the mechanisms responsible for the immunomodulatory role of ANXA1 have not been completely elucidated. Seemingly, it has been reported different results depending the experimental settings and cellular models tested (D’Acquisto et al., 2008; Yang et al., 2013).

Furthermore, ANXA1 N-terminal mimetic peptide Ac 2–26 has been proposed as a potential therapeutic strategy for cancer treatment, targeting ANXA1-mediated functions and related signaling pathways. As a proof of principle, it has been demonstrated that Ac 2–26 is able to decrease the proliferation of cervical cancer cells, through the activation of the MAPK family and targeting p53 to arrest the cell cycle (Cardin et al., 2019). It has also been showed that Ac 2–26 could be used to treat non-small cell lung cancer (NSCLC), regulating NFkB pathway (Guan et al., 2019). Remarkably, this ANXA1 mimetic has demonstrated cardioprotective actions against *in vivo* myocardial infarction, thereby effectively reducing cardiac inflammation, fibrosis and apoptosis (Qin et al., 2019). Fredman and coworkers designed nanoparticles coupled to Ac 2–26 to target collagen IV as a treatment for advanced atherosclerotic lesions, which caused a reduced lesion instability in an FPR2-dependent manner (Fredman et al., 2015). Hence, the combination of Ac 2–26 with nanoparticles emerges as a useful and promising therapeutic approach for cancer treatment.

### Clinical and biological roles in HNC

A pioneer study led by our research group demonstrated the downregulation of ANXA1 protein expression in HNSCC tissue specimens by both Western blot analysis and immunohistochemistry (Garcia Pedrero et al., 2004). The loss of ANXA1 expression was significantly associated with poor prognostic parameters, i.e., larger tumors, locoregional metastases, poor differentiation, and advanced disease stages. ANXA1 expression in HNSCC was closely related with the tumor differentiation and therefore it emerged as a differentiation marker potentially applicable for histopathological grading. In addition, our results unprecedentedly revealed the clinical utility of ANXA1 for early and accurate detection of epithelial dysplasia, since ANXA1 loss occurred in early tumorigenesis and was detected in all dysplastic precancerous lesions (Garcia Pedrero et al., 2004). We also further contributed to uncover a transcriptional regulatory mechanism underlying ANXA1 downregulation in HNSCC. We thus found that miR-196a/b levels inversely correlated with ANXA1 expression in paired HNSCC tissue samples and that ANXA1 was a direct target of these miRNAs (Álvarez-Teijeiro et al., 2017a; Álvarez-Teijeiro et al., 2017b).

**FIGURE 3:**
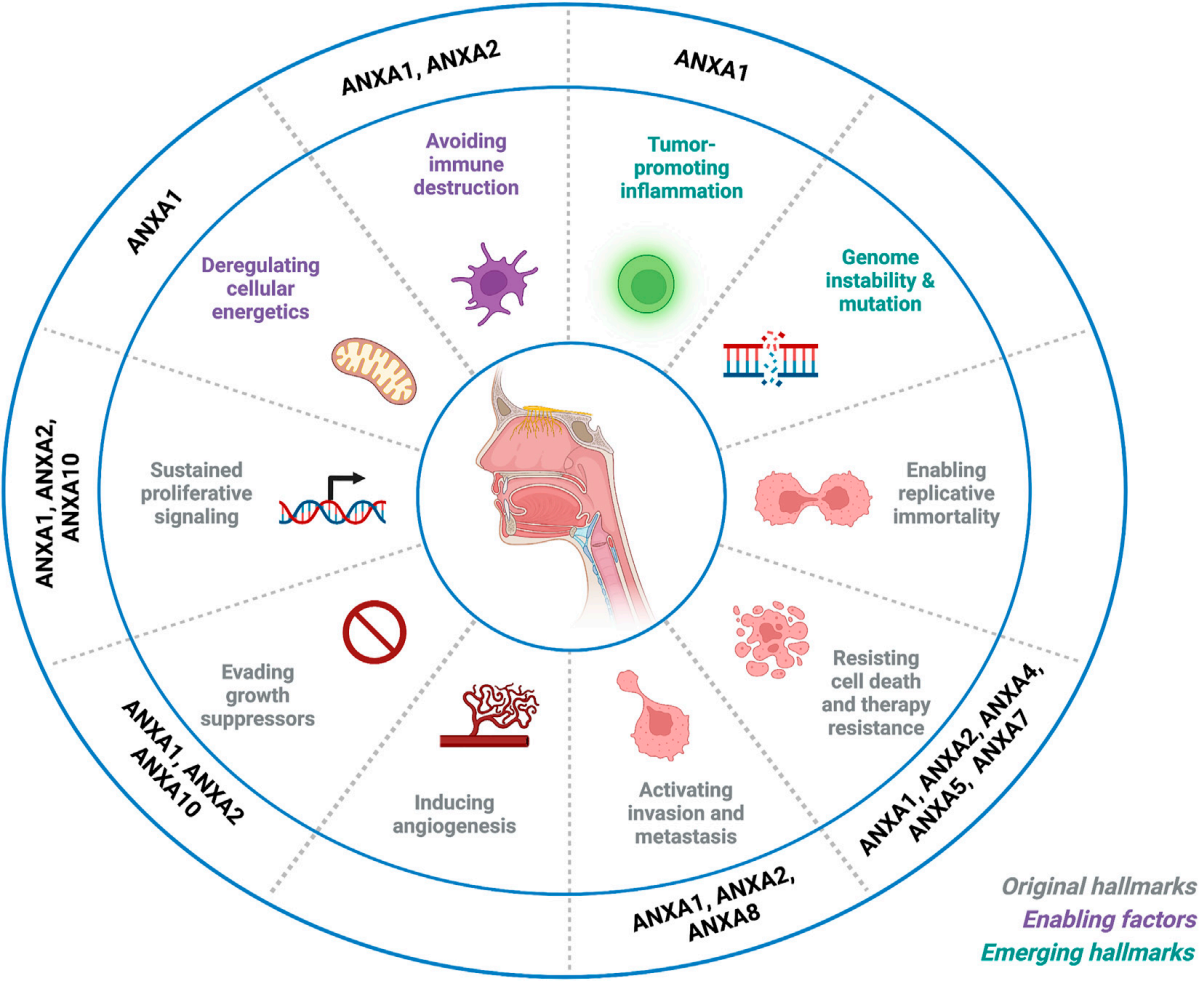
Impact of annexin dysregulation on the hallmarks of head and neck cancer. Schematic representation that summarizes current knowledge on annexin dysregulation in relation to the hallmarks of cancer defined by Hanahan and Weinberg. Image created with BioRender.com.

Studies from other groups have confirmed ANXA1 down-regulation in HNSCC, and further contributed to functionally and mechanistically characterize the specific role of ANXA1 in the progression of these tumors. Thus, ANXA1 expression has been linked to the regulation of different cellular processes in HNC thereby affecting multiple hallmarks of cancer (Figure 3). It has been reported that ANXA1 expression inversely correlates with EGFR, and it regulates the intensity and duration of the EGFR-dependent signaling events and the exosome phopho-EGFR release affecting cell-cell communication (Raulf et al., 2018). In NPC, ANXA1 was found to compete with Cbl for binding to EphA2, increasing its stability by inhibiting EphA2 ubiquitination and degradation mediated by Cbl. Consistently, ANXA1 binding to EphA2 increased EphA2 levels and its oncogenic activity, enhancing tumor growth and metastatic dissemination *in vitro* and *in vivo*. Moreover, patients with high expression of both proteins showed a poorer disease-free survival and overall survival compared to patients with high expression of only one protein (Feng et al., 2021).

ANXA1 also regulates autophagy in NPC cells through the inhibition of the proteins BECN1 and ATG5, promoting cell migration, invasion and metastasis. ANXA1-mediated autophagy suppression involves the PI3K/Akt signaling pathway (Zhu et al., 2018). Likewise, ANXA1 can also exert a pro-tumorigenic role in HNC by regulating tumor growth and metastasis through FPR2 (Gastardelo et al., 2014). ANXA1 has also been linked to radio- and chemoresistance (Zhuwang et al., 2013; Suh et al., 2015). Moreover, ANXA1 mRNA levels were found to diminish in OSCC patients compared to controls and detectable in liquid biopsy, postulating its application as a blood-based biomarker (Faria et al., 2010).

## Annexin A2

### Structural and functional characteristics

Annexin A2 (ANXA2; also known as lipocortin II or calpactin-1 heavy chain) is a peripheral membrane-binding protein encoded by *ANXA2* gene, located at 15q22.2. There are three pseudogenes located on chromosomes 4, 9, and 10. ANXA2 protein is mainly expressed at the surface of several cell types, including epithelial cells, macrophages and mononuclear cells (Hedhli et al., 2012; Wang and Lin, 2014). It can be detected as a monomer, heterodimer or heterotetramer (Waisman, 1995). It is mainly found as a multifunctional heterotetrametric form composed by two subunits of ANXA2 bridged non-covalently with a S100A10 dimer (A2t or AIIt) (Thiel et al., 1992; Deora et al., 2004). It results in an enhanced binding affinity to membrane phospholipids; consequently, this complex is mainly found associated with the plasma membrane and specific membrane-bound structures. Its subunits contains three type II and two type III Ca (2+)-binding sites, being able to mediate the interaction between ANXA2 complex AIIt (S100A10 and S100A11) and anionic phospholipid, F-actin, and heparin (Liemann and Lewit-Bentley, 1995; Liemann and Huber, 1997; Filipenko and Waisman, 2001). Phosphorylation at tyrosine residue Tyr23 induces nuclear translocation, while phosphorylation of the serine residues Ser11 and Ser25 allows ANXA2 export from the nucleus (Liu J. et al., 2003).

ANXA2 participates in several cellular functions in both health and disease. It plays a prominent regulatory role of membrane trafficking, and actin-related membrane dynamics. ANXA2/S100A10 heterotetramer formed by Ca2+ presence binds to cortical actin network, regulating exocytosis (Creutz, 1992), micropinocytosis (Merrifield et al., 2001), and plasmatic membrane cytoarchitecture (Benaud et al., 2004). The heterotetrameric complex plays a role in the subcellular distribution of early and recycling endosome flow process (Harder and Gerke, 1993; Zobiack et al., 2003), whereas it is suggested that ANXA2 monomeric form has been related to cholesterol-mediated endocytosis (Morel and Gruenberg, 2007).

ANXA2 has also been implicated in the maintenance of fibrinolytic homeostasis by its interaction with tenascin-C and the tissue-type plasminogen activator on the cell surface, which is crucial for the degradation of fibrin (Gerke et al., 2005).

### Altered expression and pathobiological role in cancer

ANXA2 dysregulation is a common feature in multiple cancers (Li Z. et al., 2021). Similar to ANXA1, ANXA2 may elicit either tumor-promoting or -suppressive mechanisms depending on the cancer type; however, ANXA2 is frequently enhanced in metastatic cancers. ANXA2 expression has been shown to promote proliferation, invasion and metastasis in gliomas (Zhai et al., 2011), ovarian cancer (Lokman et al., 2013), hepatomas (Zhang et al., 2013), breast cancer (Sharma et al., 2006) and pancreatic cancer (Zheng et al., 2011). Conversely, there are tumors where ANXA2 acts as a tumor suppressor. In prostate cancer, ANXA2 expression is reduced or lost in cell lines, and its overexpression inhibited cell migration (Liu J. W. et al., 2003). ANXA2 is also found downregulated in ESCC and significantly correlated with lymph node metastasis and pathological differentiation (Feng et al., 2012). In osteosarcomas, increased protein expression was correlated with lower metastatic potential (Gillette et al., 2004).

ANXA2 regulation by post-translational modifications plays a critical role. In particular, phosphorylation on serine residues Ser11, Ser25 and tyrosine Tyr23 may have important functional implications in a variety of cellular processes, which are also relevant for tumor cell biology as major hallmarks of cancer. Phosphorylation of Tyr23 residue on ANXA2 has been pointed as a key regulator of cell motility. Upon Tyr23 phosphorylation, ANXA2 binds to actin filaments to enhance or inhibit migration modulating cytoskeletal structure (de Graauw et al., 2008; Kpetemey et al., 2015).

Tyr23 phosphorylation may promote cell migration by regulating the DOCK3/β-catenin/WAVE2 axis in different models of hepatoma cells (Cui et al., 2016). It has also been reported that Tyr23 phosphorylation triggers epithelial-to-mesenchymal transition (EMT) mediated by Rho, transforming growth factor (TGF)-β and Twist/Snail pathways (Rescher et al., 2008; Zheng et al., 2011; Chen et al., 2015).

ANXA2 has been reported as a major cellular substrate of Src that phosphorylates ANXA2 on Tyr23 regulating actin interactions and subsequently cell migration (Shetty et al., 2012). Based on this, a recent report by Mahdi et al., 2020 demonstrated EGF-dependent ANXA2 phosphorylation on Tyr24, which was assumed but not demonstrated to be by Src (Mahdi et al., 2020). In addition, ANXA2 promotes cell invasion mediated by Src/ANXA2/STAT3 pathway (Rocha et al., 2018). Similarly, YES1 phosphorylates ANXA2 on Tyr24, which drives gastric cancer invasion and metastasis by the activation of EphA2/YES1/ANXA2 axis (Mao et al., 2021).

ANXA2 is indirectly involved in Akt pathway regulation. When cells are exposed to irradiation, Akt protein binds to the heat shock protein 27 (HSP27), which ameliorates the effects on DNA damage and apoptosis by radiation (Kanagasabai et al., 2010).

ANXA2 influences p53 level by activating JNK/c-Jun signaling and suppressing p53 expression and its downstream target genes, p21, BAX and GADD45, which regulate apoptosis (Wang et al., 2012; Feng et al., 2017). It is well-known that nutrition and oxygen are indispensable for tumor cells to grow and survive. So, in the early stage of tumor starvation, ANXA2 may support starving cells by inducing autophagy (Moreau et al., 2015).

There are also evidences for a potential application of ANXA2 as a therapeutic target. Ricciardelli et al. used an anti-ANXA2 antibody to reduce both tumor growth and metastasis in an ovarian cancer mice model (SK-OV3) (Lokman et al., 2013). One year later, Mandip Singh and others inserted short hairpin (sh)RNA targeting ANXA2 (shANXA2) into a cationic ligand-guide (CLG, a liposomal carrier) to construct a CLG-ANXA2 compound. The CLG-ANXA2 was designed to recognize cancer cells and CSCs in a lung cancer mouse model (H1650). After CLG-ANXA2 was taken up by tumor cells, shANXA2 reduced ANXA2 mRNA and protein levels. The CLGshANXA2 subgroup showed reduced tumor growth (72–75% relative to the control) (Andey et al., 2014). It has also been recently described an ANXA2-targeting peptide motif CBP12 with highly selectivity and affinity to ANXA2 and proved ability to specifically target colorectal cancer cells, therefore emerging as a candidate for ANXA2-targeted therapeutic strategies (Staquicini et al., 2017). Interestingly, listeria-based anti-ANXA2 targeting vaccine (Lm-ANXA2) in combination with anti-PD-1 antibodies have demonstrated to be effective in PDAC mice models (Kim et al., 2019).

Noteworthy, ANXA2 gene silencing has demonstrated to downregulate the expression of proangiogenic proteins including vascular endothelial growth factor as (VEGF)-R2, VEGF-C, matrix metalloproteinases such as MMP-2, MMP-9, MT1-MMP and also the metalloproteinase inhibitor TIMP-2 (Bao et al., 2009).

ANXA2 may play an immunomodulatory role, since ANXA2 interacts with dendritic cell (DC)-specific intracellular adhesion molecule (ICAM)-3 grabbing non-integrin (DC-SIGN, CD209) leading to immunosuppression (Chao et al., 2015). This suppression might influence the clinical efficacy and outcome of anticancer therapies.

ANXA2 has also been linked to treatment resistance. Intracellular ANXA2 binds the p50 subunit of nuclear factor (NF)-κB by exposure of pancreatic cancer cells to genotoxic agents. This complex is translocated to the nucleus to activate the NF-κB signaling pathway, which modulates cell apoptosis and drug resistance (Jung et al., 2015). Nuclear translocation of ANXA2 also exhibited a protective effect against DNA damage caused by irradiation in human lung and breast cancer cells (Madureira et al., 2012; Grindheim et al., 2016).

Besides, it has been described a reciprocal regulation between ANXA2 and ERG oncoprotein in prostate cancer (Griner et al., 2015) and with HER2 in breast cancer (Shetty et al., 2012)

### Clinical and biological roles in HNC

There are controversial findings on the function of ANXA2 in HNSCC. Different studies have reported relationships of ANXA2 with poor or good prognosis depending on the tumor location or the studied cohort. These discrepant results could be related to differences on patient characteristics, such as race (Beebe-Dimmer and Cooney, 2019), smoking (Bezerra et al., 2018), oral health habits (Kawakita et al., 2017), HPV infection (Hübbers and Akgül, 2015), etc.

ANXA2 is upregulated in several tumor subtypes but in HNSCC is usually found downregulated, and low ANXA2 levels associated with poorly differentiated tumors and metastasis in OSCC, laryngeal/pharyngeal squamous cell carcinoma (Pena-Alonso et al., 2008; Rodrigo et al., 2011b, 2014). Also, intestinal-type sinonasal adenocarcinomas with loss of expression of ANXA2 are associated to the more aggressive histopathological types (Rodrigo et al., 2011a). By contrast, another study reported high ANXA2 protein expression in OSCC associated with disease stage, differentiation grade, lymph node metastasis and poor patient survival (Ma and Wang, 2021). This study also found that ANXA2 silencing by siRNA was able to significantly reduce the proliferation, migration and invasion of OSCC cells.

Some studies have associated high ANXA2 expression levels to poor overall survival and disease-free survival, and advanced disease status in esophageal carcinoma (Ma et al., 2014), and NPC (Chen et al., 2015). Notably, ANXA2 participates in facilitating host cell infection by HPV16 resulting in a high risk of tumor progression (Woodham et al., 2012). Moreover, ANXA2 has been linked to resistance to radio and chemotherapy in NPC (Chen et al., 2015), while ANXA2 interaction with dendritic cells caused immunosuppression mediated by IL-10 production (Chao et al., 2015).

In summary, various ANXA2-targeted therapeutic strategies have already been developed and tested, demonstrating antitumor efficacy in some preclinical cancer models. However, despite widespread dysregulation of ANXA2 expression in different cancers, the biological and clinical impact of ANXA2 targeting may considerably vary depending on cellular and tissue specificity, Therefore, a potential clinical application will require individual testing and deep functional and mechanistic characterization for each cancer type. Furthermore, ANXA2 role modulating radio and chemotherapy as well as immune response exposes it as a combination target to overcome resistance and to improve treatment response.

## Annexin A3

Annexin A3 (ANXA3), also known as lipocortin III or placental anticoagulant protein III, is a calcium-dependent phospholipid-binding protein that affects membrane trafficking and organization (Gerke and Moss, 2002). It has two isoforms that vary slightly in their molecular weights (33 and 36 kDa) (Moss and Morgan, 2004), result from the alternative splicing of exon 3 within the *ANXA3* gene (Bianchi et al., 2010) located at chromosome region 4q13-q22 (Tait et al., 1991). This protein contains two special tryptophan residues, which can influence its interaction with phospholipid membranes (Sopkova et al., 2009). Regarding its functions, ANXA3 was first shown to inhibit PLA2 (Coméra et al., 1990) and to cleave the cyclic bond of inositol 1,2-cyclic phosphate to form inositol 1-phosphate (Ross et al., 1990). Nevertheless, later studies have confirmed its involvement in a wide variety of biophysiological processes, including vesicular transport (Faugaret et al., 2011), cell migration (Zhang Z. et al., 2021), angiogenesis (Park et al., 2005), apoptosis (Chong et al., 2010), inflammation (Meng et al., 2019) or sepsis (Toufiq et al., 2020) among others.

Similar to other members of the annexin family, aberrant expression of ANXA3 has been linked to cancer pathogenesis, although it is highly dependent on the tissue of origin. Accordingly, ANXA3 expression has been found upregulated in breast, colorectal or pancreatic tumors, while downregulated levels have been detected in renal, thyroid or prostate cancers. Research performed assessing the functional role of ANXA3 in cancer has demonstrated its involvement in vital processes for tumor development and progression (Liu C. et al., 2021; Yang et al., 2021), such as sustaining proliferative signals from ERK and JNK pathways in colorectal (Xu R. et al., 2019) and hepatocellular carcinomas (Tong et al., 2015), or PI3K/Akt pathway in pancreatic cancer (Wan et al., 2020). In addition, aberrant ANXA3 expression has also been shown to downregulate multiple pro-apoptotic proteins, promote tumor invasion and metastasis, induce angiogenesis and even regulate drug resistance in different tumor types (Liu C. et al., 2021; Yang et al., 2021).

Regarding its clinical relevance, high ANXA3 expression has been correlated with a poor prognosis in gastric, hepatocellular and breast carcinomas (Liu C. et al., 2021), and it has also been proposed as a diagnostic marker in prostate cancer (Schostak et al., 2009).

Despite mounting evidence supporting the involvement of ANXA3 in cancer development and progression, as of yet, very little has been unraveled regarding its specific contribution to HNC. Only one study has been published in which ANXA3 was identified as a downstream target of EGFR using a functional proteomic approach in NPC-derived cells treated with TGF-α (Ruan et al., 2010). This approach confirmed that treatment of CNE2 cells with TGF-α induced tyrosine phosphorylation of ANXA3, which was blocked by treatment with the EGFR inhibitor PD153035 (Ruan et al., 2010). Even though the relevance of ANXA3 phosphorylation/dysregulation has not yet been determined, these results suggest a potential impact on NPC treatment, and encourage deepening on ANXA3 contribution in HNC since it could positively impact patient management and disease outcome.

## Annexin A4

Annexin A4 (ANXA4), also known as lipocortin IV or placental anticoagulant protein II, has 45–59% identity with other members of its family and shares a similar size and exon-intron organization (Moss and Morgan, 2004). It is a calcium and phospholipid binding protein that promotes membrane fusion and is involved in exocytosis (Oling et al., 2000; Kaetzel et al., 2001). Physiologically, ANXA4 has been shown to inhibit adenylyl cyclase 5 (Heinick et al., 2015, 2020), an enzyme that catalyzes the conversion of ATP to adenosine 3′,5′-cyclic monophosphate (cAMP), and also to interfere with sulfatide-induced plasma coagulation (Nakayama et al., 2020). ANXA4 acts as a sensor of negative membrane curvatures suggesting a role in the restoration of plasma membrane (Florentsen et al., 2021). In addition, it has been implicated in the migration of both skin fibroblasts (Wang et al., 2020) and airway epithelial progenitor cells (Jiang et al., 2018), as well as in trophoblast invasion via the PI3K/Akt/eNOS pathway (Xu Y. et al., 2019).

Accordingly, ANXA4 has also been involved in cell migration and invasion in different cancers. Thus, reduced ANXA4 levels can hamper these two processes in cells derived from gastric cancer (Ji et al., 2022), gallbladder cancer (Yao et al., 2016) and ovarian clear cell adenocarcinoma (Wang et al., 2017); meanwhile its overexpression in colorectal cancer cells was able to promote their migratory and invasive potential (Peng et al., 2021). In addition, ANXA4 expression has been associated to poor prognosis in ovarian serous carcinoma (Choi et al., 2013).

Interestingly, ANXA4 translocation from plasma membrane to cytosol has been related to resistance to paclitaxel in lung cancer cells (Gaudio et al., 2016; Scala et al., 2021) and also its upregulation has been linked to cisplatin (CDDP) resistance in mesothelioma cells (Yamashita et al., 2012), ovarian clear cell carcinoma cells (Morimoto et al., 2014) and non-small cell lung cancer (NSCLC) cell lines (Zheng et al., 2018).

Very limited information has been published to date on the role of ANXA4 in HNC. The study by Deng et al. (Deng et al., 2012) was the first to explore ANXA4 expression in laryngeal tissues. Even though the analysis was only performed in 30 patient samples, it clearly showed that its expression was frequently detected (73%) in this cancer type (Deng et al., 2012). In addition, based on a differential proteomic expression analysis, ANXA4 has been postulated as a biomarker for OSCC diagnosis and prognosis (Liu W. et al., 2016). ANXA4 expression was early detected in oral premalignant lesions (oral submucous fibrosis, OSF) as well as in the corresponding patient-matched OSCC tissue samples, where expression was found to correlate with tumor stage and poor prognosis (Liu W. et al., 2016).

Another study compared proteomic profiles in saliva samples from healthy donors and tamol chewers, which tend to form ulcers in the oral cavity (Kumar et al., 2021) predisposing to develop oral cancer (Phukan et al., 2001; Borkotoky et al., 2020). ANXA4 expression levels showed a 20.22% increase in tamol chewers versus healthy controls, which led to propose ANXA4 as a population screening marker for the early detection of OSCC in tamol consumers (lepcha et al., 2021). It has also been reported that ANXA4 could serve to distinguish between two different types of benign tumors of the salivary glands, Warthin’s tumors (WT) and pleomorphic adenomas (PA), as PAs differentially express proteins associated with cell death, apoptosis and tumorigenesis, like ANXA4 and ANXA1 (Donadio et al., 2013). Moreover, it has been suggested a possible contribution of ANXA4 to OSCC chemoresistance, because this protein was found differentially expressed among others, in Sirt1-induced resistance to CDDP (Xiong et al., 2011). However, further investigation is needed to demonstrate a direct participation in OSCC chemoresistance, as well as to extend ANXA4 analysis to other HNSCC subsites.

Together these data emphasize the conceivable application of ANXA4 as a biomarker for OSCC diagnosis and prognosis, but also therapeutically, since targeting ANXA4 expression might hinder early tumorigenesis, progression and management of OSCC, and perhaps other HNC types.

## Annexin A5

Annexin A5 (ANXA5) is the smallest member of the family with 36 kDa. Similar to ANXA4, it plays an important role in membrane repair (Bouter et al., 2015). However, probably the most known function of ANXA5 is its ability to bind PS, which is rapidly mobilized from the inner to the outer side of the membrane in cells undergoing apoptosis or necrosis (Rysavy et al., 2014). This characteristic together with the fact that ANXA5 is not toxic or immunogenic when used *in vivo* has made this protein a valuable tool for *in vivo* imaging of cell death processes (Chopra et al., 2012).

In the context of cancer, high ANXA5 expression has been associated with poor outcome in gliomas (Zhong et al., 2021), hepatocarcinomas (Sun et al., 2018), bladder (Wu et al., 2021), colorectal (Xue et al., 2009) and cutaneous squamous cell carcinomas (Shapanis et al., 2021). Interestingly, ANXA5 levels were higher in bladder cancer patients with lymph node involvement (Mitra et al., 2006), which is consistent with studies performed on cellular models of hepatocarcinoma. The authors demonstrated that the loss of ANXA5 expression decreased cell adhesion to lymph nodes, and also reduced the formation of lymph node metastasis *in vivo* (Sun et al., 2018). In addition, *in vitro* experiments have shown that ANXA5 depletion may hamper proliferation, migration, invasion and metastasis formation in various tumor cells from breast cancer (Bouvet et al., 2020), uterine cervical carcinoma (Li et al., 2018), hepatocarcinoma (Sun et al., 2018) and cholangiocarcinoma (Ding et al., 2017). In marked contrast to these data, it has been reported that positive ANXA5 expression correlates with a better prognosis in adult acute myeloid leukemia (AML) (Niu et al., 2019), and a tumor suppressor role has been described in gastric cancer cells through the repression of ERK pathway (Wang et al., 2021).

On the other hand, it has also been reported that PS is expressed in the surface of cancer cells and also vascular endothelial cells in tumors, whereas it cannot be detected in normal endothelium (Ran and Thorpe, 2002). Therefore, the ability of ANXA5 to bind to PS could be exploited for therapeutic purposes in cancer patients. According to this purpose, it has been demonstrated that ANXA5 may improve specific cancer cell targeting when combined with different therapeutic strategies, such as phototermal nanotubes (McKernan et al., 2021), vaccine antigens (Kang et al., 2020), or enzyme-prodrug systems (Virani et al., 2018). In all cases, treated mice experienced a prolonged survival without any noticeable toxic effects in healthy tissues (Virani et al., 2018; Kang et al., 2020; McKernan et al., 2021). Furthermore, ANXA5 administration in mice enhanced immunogenicity by binding to PS and inducing systemic cytotoxic T-cell responses, leading to tumor regression and reduced relapse (Li et al., 2020). It has also proved to rescue immune suppression after chemotherapy, thus contributing to generate a robust antitumor immunity (Kang et al., 2020).

In the specific context of HNC, ANXA5 was found overexpressed in 80% of the laryngeal cancer samples analyzed, although there is no available information on the clinical significance of this finding (Deng et al., 2012). Through a proteomic profiling approach, ANXA5 overexpression was also detected in malignant major salivary gland tumors (MSGT) versus the adjacent healthy tissue (Seccia et al., 2020); however, significant correlations between protein expression and the clinical parameters were not observed (Seccia et al., 2020). In a meta-analysis of gene expression in public datasets, ANXA5 was found among other 95 genes whose expression had a significant impact over OSCC patients’ survival (Bajrai et al., 2021). These data suggest that ANXA5 might play a role in OSCC progression, although a more in-depth analysis is needed to clarify and validate this finding. In addition, a comparative protein expression analysis in NPC-derived cells revealed that ANXA5 levels were increased in cis-diamminedichloroplatinum (cDDP)-resistant cells compared to the parental CNE2 cells (Tang et al., 2012). These results suggest that ANXA5 could contribute to cDDP resistance in NPC; however, more experiments are required to confirm its direct involvement, and to extend analysis to other HNC types.

These data evidence that the role of ANXA5 in HNC development and progression remains largely unknown. Therefore, more efforts are needed to unravel this, in particular, its therapeutic potential due to the high affinity of ANXA5 for PS. On this basis, ANXA5 targeting could emerge as a valuable strategy to counteract immune evasion in HNC, and ultimately contribute to trigger a potent antitumor immune response (Birge et al., 2016).

## Annexin A6

Annexin A6 (ANXA6) is the only protein from this family structurally comprising 8 annexin repeats that form two cores in the C-terminal domain (Qi et al., 2015). This peculiarity may render specific membrane-interaction mechanisms (Buzhynskyy et al., 2009). ANXA6 is expressed in most human tissues, and mainly localized in plasma membrane and endosomes (Grewal et al., 2017). Physiologically, ANXA6 plays a crucial role as a scaffold protein and in the organization of membrane domains, where it interacts with many different partners into multifactorial complexes that orchestrates different signaling pathways. As a result, ANXA6 is involved in many events associated with membrane organization, such as cholesterol homeostasis, interactions with actin cytoskeleton proteins, regulation of endocytic trafficking and secretory events, modulates calcium flux and homeostasis and is involved in the reorganization of cytoskeleton during cell migration. In relation to its scaffolding properties, ANXA6 regulates several signaling pathways such as EGFR/Ras/MAPK and FAK/PI3K (Qi et al., 2015; Grewal et al., 2017). ANXA6 has been directly involved in regulating Ca2+ entry. Its stable membrane expression reduces calcium store-operated entry and reduces cell proliferation. Its interaction with plasma membrane and subsequent cortical actin stabilization attenuates calcium entry *in vivo* (Monastyrskaya et al., 2009). ANXA6 has also been involved in membrane repair mechanisms (Potez et al., 2011; Swaggart et al., 2014), inducing constriction forces and acting synergistically with ANXA4 (Boye et al., 2017).

ANXA6 has been involved in several biological processes such as cell proliferation, survival, membrane repair, migration and adhesion (Grewal et al., 2017), which are often dysregulated in cancer. In most cases, ANXA6 acts as a tumor suppressor, but some oncogenic roles have also been described depending on the cancer type and disease stage.

It has been demonstrated that ANXA6 present in extracellular vesicles may play a role in cancer. In pancreatic ductal adenocarcinoma (PDA), cancer-associated fibroblasts (CAF)-derived extracellular vesicles containing the complex ANXA6/LRP1/TSP1 enhanced tumor aggressiveness, and ANXA6 was crucial to promote aggressive phenotypes, invasion *in vitro*, and metastasis formation *in vivo* (Leca et al., 2016). Exosomal ANXA6 has also been involved in chemotherapy resistance. Exosomal ANXA6 from gemcitabine-resistant cells induced gemcitabine resistance in sensitive triple negative breast cancer (TNBC) cells, at least by downregulating EGFR (Li T. et al., 2021). Another study from Uchihara et al. showed that ANXA6 from CAF-derived extracellular vesicles induced drug resistance in gastric cancer cell lines via integrin beta1 stabilization in the cell surface and subsequent FAK-YAP pathway activation (Uchihara et al., 2020). In breast cancer, ANXA6 present in exosomes from stem cells promoted paclitaxel resistance via YAP1 upregulation. ANXA6 also promoted cell migration, autophagy, cell growth and stem properties (Guo et al., 2021). In addition, ANXA6 has been involved in resistance to EGFR-TKIs such as lapatinib in TNBC, where ANXA6 upregulation is part of an adaptive mechanism to acquire resistance (Widatalla et al., 2019). Another study from the same group showed that ANXA6 could also promote resistance to some EGFR-TKIs in breast cancer cells (Koumangoye et al., 2013).

ANXA6 has been extensively studied in breast cancer. ANXA6 is required for the cell-cell and cell-ECM contacts, and its loss contributes to tumor progression by promoting loss of cell contacts and anchorage-independent growth. ANXA6 was also found necessary for an efficient motility and invasion of breast cancer cells. Sakwe et al. discussed these apparent discrepancies and expound the varying levels of ANXA6 expression across breast cancer development (Sakwe et al., 2011). Hence, ANXA6 may function as a tumor suppressor or tumor promoter, depending on the cancer subtype and degree of malignancy. ANXA6 antitumor role may be more relevant in TNBC than in non-triple negative breast cancers (Korolkova et al., 2020b). The involvement of ANXA6 in plasma membrane repair may allow cancer cells to rapidly respond to small membrane injuries that arise frequently, as demonstrated in MCF-7 cells (Boye et al., 2017). Moreover, a reciprocal regulation between ANXA6 and the Ca2+ activated RasGRF2 (GRF2) has also been recently reported in TNBC, which could serve to distinguish rapidly growing tumors from those more aggressive and highly invasive (Korolkova et al., 2020a).

Ectopic expression of ANXA6 in the human A431 squamous epithelial carcinoma cells reduced tumor growth suggesting an antitumor role for ANXA6 in A431 cells (Theobald et al., 1995). ANXA6 overexpression also reduced LDL cholesterol-induced migration and invasion of A431 cells (Jose et al., 2022). Interestingly, ANXA6 reduced A431 cell migration and invasiveness, and enhanced EGFR-TKIs-mediated inhibition of growth and migration in these cells (Hoque et al., 2020)*.* All the aforementioned studies highlight the anti-tumor role of ANXA6 in A431 squamous epithelial cells overexpressing EGFR. Given that EGFR overexpression is a common feature in HNSCC, an analogous role for ANXA6 could be plausible, which merits further investigation.

Several studies have demonstrated that ANXA6 is a scaffold protein that modulates EGFR/Ras/MAPK pathway. Thus, ANXA6 interacts with several proteins like p120 GAP, Raf1 and PKCalfa, downregulating this pathway, thereby acting as a tumor suppressor in several human cancers (Pons et al., 2001; Grewal et al., 2005; Muga et al., 2009; Cornely et al., 2011; Koese et al., 2013; Wang et al., 2013). This is a very important mechanism in HNSCC, where ANXA6 promotes PKCalfa-mediated EGFR inactivation, acting as a scaffold for PKCalfa and promoting its recruitment to plasma membrane and its interaction with EGFR. ANXA6 also promoted PKCalfa-dependent inhibitory feedback over EGFR pathway. It is also important to mention that downregulation of ANXA6 expression occurs in several malignancies with EGFR upregulation, maybe as a possible mechanism to prevent the antitumor role of ANXA6 (Koese et al., 2013). By contrast, it has been described a protumor role for ANXA6 and its involvement in drug resistance (Koumangoye et al., 2013; Widatalla et al., 2019), related to its ability to modulate biological membranes, and to stabilize activated receptors in the membrane (Cornely et al., 2011), such as EGFR (Koumangoye et al., 2013).

ANXA6 expression has been proposed as a useful biomarker in several human cancers. ANXA6 acts as a tumor suppressor in cervical cancer via autophagy induction *in vitro* and *in vivo*. Besides, ANXA6 expression levels may serve as a predictive biomarker of survival for cervical cancer patients (Sun et al., 2020). Nuclear ANXA6 expression has been proposed as a protein marker for squamous cervical cancer diagnosis (Lomnytska et al., 2010), which may improve cervical cancer diagnosis at early stages and patient monitoring (Lomnytska et al., 2011). ANXA6 has also been included as a serum biomarker in a 4-protein panel for esophageal adenocarcinoma detection (Zaidi et al., 2014). ANXA6 is downregulated in human hepatocellular carcinoma, and suggested as a putative biomarker for these tumors (Meier et al., 2016). In basal-like breast cancer, reduced ANXA6 expression was significantly associated with higher recurrence-free but lower distant metastasis-free and overall survival. Koumangoye et al. also proposed ANXA6 expression as a biomarker to identify patients likely to respond to EGFR-TKIs (Koumangoye et al., 2013). In addition, ANXA6 has emerged as a potential biomarker in ovarian cancer, since it was found significantly upregulated in tumor tissue samples, particularly in stages II-IV compared to health tissue (Noreen et al., 2020). High ANXA6 expression has been associated with a poor survival in PDA, and ANXA6 levels in circulating extracellular vesicles (EVs) postulated as a diagnostic and prognostic biomarker in PDA (Leca et al., 2016). Similarly, serum exosomal ANXA6 levels could serve as a predictive biomarker of response to gemcitabine-based chemotherapy in TNBC patients (Li T. et al., 2021).

## Annexin A7

Annexin A7 (ANXA7), also known as synexin, presents a long hydrophobic N-terminal domain, rich in glycine, tyrosine and proline. This protein is predominantly located in plasma membrane, secretory vesicles and nuclear envelope (Guo et al., 2013a; Grewal et al., 2016). ANXA7 is deeply involved in calcium homeostasis, and it was actually the first annexin shown to function as a calcium channel. It is involved in exocytic secretion and aggregation of chromatin granules. It also displays a GTPase function, interacting and hydrolyzing GTP in several cellular processes, such as calcium/GTP-dependent exocytic trafficking (Gerke and Moss, 2002; Grewal et al., 2016). Besides its functions in calcium homeostasis and as a GTPase, ANXA7 negatively regulates COX-dependent prostaglandin (PGE_2_) production and is also involved in cardiac remodeling, as well as in cell proliferation regulation (Grewal et al., 2016).

ANXA7 has also been reported to play a role in cancer, either oncogenic or oncosuppressor depending on the tumor subtypes. As discussed below, ANXA7 may act as an oncogene in gastric cancer, hepatocellular carcinoma, nasopharyngeal carcinoma and breast cancer; or as an oncosuppressor in melanoma, prostate cancer, and glioblastoma.

In gastric cancer, the significance of ANXA7 expression remains controversial. Loss of ANXA7 expression has been associated with distant metastases in gastric cancer, hence suggesting an oncosuppressor role (Hsu et al., 2008). However, other studies have pointed to a protumor role of ANXA7 in gastric cancer. Yuan et al. demonstrated that high ANXA7 expression was an independent predictor of poor survival, which was associated with poor differentiation, and presence of lymph node metastasis (Yuan et al., 2014). ANXA7 expression was also found to be downregulated along gastric cancer progression and inversely correlated with apoptosis (Ye et al., 2018). These findings could reflect an anti-apoptotic role for ANXA7 in gastric pathogenesis and a potential biomarker for diagnosis, prognosis and treatment for gastric adenocarcinoma patients (Ye et al., 2018).

ANXA7 upregulation in hepatocellular carcinoma has been associated with enhanced invasion and lymphatic metastasis (Sun et al., 2009b; 2009a). In Her2-negative breast cancer, ANXA7 expression correlated with metastasis and low survival rate, and serves as a diagnostic and prognostic biomarker for these patients (Srivastava et al., 2004). In addition, Srivastava et al. showed that high expression of ANXA7 was a strong predictor of reduced disease-free survival (Srivastava et al., 2001a), and thus highlights its potential as a prognostic biomarker to predict breast cancer patients’ survival.

On the other hand, supporting a tumor-suppressor role, ANXA7 has been pointed as a marker for a less invasive phenotype in melanoma (Kataoka et al., 2000). ANXA7 also exhibits a suppressive role in prostate cancer. ANXA7 inhibits tumor growth and cell proliferation, and its loss of expression has been correlated with late-stage prostate cancer (Srivastava et al., 2001b). Moreover, reduced ANXA7 expression occurred significantly in metastatic and hormone refractory prostate cancer compared to benign hyperplasia (Srivastava et al., 2001a).

ANXA7 expression has been reported an independent outcome predictor in glioblastoma multiforme, and its expression correlated with longer survival in patients with GBM (Hung and Howng, 2003). ANXA7 has been shown to downregulate EGFR in glioblastoma and loss of ANXA7 mRNA expression was associated with poor survival and prognosis in glioblastoma patients. Altogether, these data support an oncosuppressive role of ANXA7 in these malignancies as well as an oncogenic synergistic effect between ANXA7 loss and EGFR amplification (Yadav et al., 2009).

In the context of HNC, it has only been reported that ANXA7 silencing enhanced radiosensitivity in NPC via apoptosis promotion (Gui et al., 2020).

## Annexin A8

Annexin A8 (ANXA8), similar to other members, plays an important role in the organization of membrane domains, especially those that constitute sites of membrane-cytoskeleton interactions, for instance, by binding F-actin and certain phospholipids such as PtdIns(4,5)P2 (Goebeler et al., 2006). Moreover, ANXA8 is highly involved in the regulation of intracellular membrane trafficking and regulation of endocytosis (Goebeler et al., 2008). Related to this latter role, ANXA8 has been implicated in the adhesion of leukocytes to the endothelium, by modulating CD63 sorting and efficient membrane presentation (Poeter et al., 2014). ANXA8 also regulates adhesion to ECM proteins like integrin β1 (Heitzig et al., 2017). All these features make ANXA8 an important regulator of key cellular processes such as migration, invasion and adhesion, often commonly dysregulated in cancer. ANXA8 mainly acts as a tumor-promoting gene in human cancers. ANXA8 upregulation was first described in promyelocytic leukemia, harboring a PML-RARA fusion, in which dysregulation of RARA gene caused such overexpression (Chang et al., 1992).

Its biological relevance has been intensely studied in mammary gland development and breast cancer. ANXA8 is regulated by all-trans retinoid acid (RA) and RA-RARA-ANXA8 axis enhances a loop of aberrant morphogenesis, rendering an abnormal mammary gland structure (Rossetti and Sacchi, 2019). In fact, ANXA2 downregulation and ANXA8 upregulation were jointly sufficient to create abnormal ductal carcinoma *in situ* (DCIS) acinar-like structures, which resemble early breast cancer lesions. Moreover, ANXA8 upregulation is detected in DCIS compared to atypical ductal hyperplasia and normal mammary gland, and also highly upregulated in ER-negative tumors compared to ER-positive ones. In addition, ANXA8 expression significantly correlated with features of breast cancer progression such as tumor grade, stage and lymph node infiltration, arising as a putative biomarker to identify ER-negative basal-like breast cancers (Rossetti et al., 2016).

Meanwhile, ANXA8 overexpression in gastric cancer has been correlated with disease stage and differentiation grading, and it emerged as an independent predictor of worse OS and DFS and a potential poor prognosis biomarker for gastric cancer patients (Ma et al., 2020). ANXA8 has also been found upregulated in squamous cell carcinoma of the uterine cervix (Chao et al., 2006). Mechanistically, ANXA8 inhibition by miR-185-3p reduced the proliferation of cervical cancer cells (Zhang and Han, 2021).

In pancreatic cancer, ANXA8 overexpression was also associated with higher histological grades and a lower survival in I-II stage patients, thereby emerging as a poor prognosis biomarker for early stages of pancreatic cancer (Pimiento et al., 2015). Another study from Karanjawala et al. found ANXA8 overexpression in infiltrating ductal pancreatic adenocarcinomas compared to normal ducts, suggesting its possible use as a diagnostic biomarker (Karanjawala et al., 2008). There are also evidences for a protumor role of ANXA8 in bladder cancer. Expression was consistently found upregulated in bladder tumors and derived cell lines and promoted tumor growth and metastases *in vitro* and *in vivo*, while ANXA8 silencing reduced tumor growth, migration, invasion and EMT (Yuan et al., 2021).

In ovarian cancer, ANXA8 expression was also found to increase during tumor progression. High ANXA8 levels were significantly associated with advanced stages, differentiation grade and nodal metastases, and proposed as a poor prognosis biomarker in epithelial ovarian cancer (Zhu et al., 2020). ANXA8 mRNA levels were also upregulated and correlated with poor overall survival and progression-free survival in patients with ovarian serous tumors. Immunohistochemical ANXA8 expression in malignant ovarian tumors correlated with FIGO stages and tumor progression, and it was revealed as an independent predictor of outcome and survival and a powerful poor prognosis biomarker for these tumors (Gou et al., 2019).

Besides, it has been demonstrated that ANXA8 regulates VEGFR-driven angiogenesis, showing a crucial role for sprouting, invasion and adhesion of human umbilical vein endothelial cells (HUVECs) to ECM proteins (Heitzig et al., 2017). Given such implications, to deepen investigation on a plausible role of ANXA8 as a therapeutic target for anti-angiogenic strategies should be encouraged.

Despite all the aforementioned studies strongly supporting a relevant role for ANXA8 in numerous cancer types, it has so far been poorly investigated in HNC. A study from Kudo et al. reported ANXA8 upregulation in maxillary squamous carcinomas harboring *TP53* mutation (Kudo et al., 2017). It has also been published that ANXA8 mRNA expression levels could serve as a biomarker to detect OSCC lymph node metastases that were histopathologically undetectable (Oka et al., 2016).

## Annexin A9

Annexin A9 (ANXA9) was first discovered in 1999 (Morgan et al., 1999a). Its structure is similar to ANXA2 and displays certain conserved domains; however, ANXA9 is an atypical annexin member, unable to bind calcium since its sequence lacks the acidic cap residue in the calcium binding domains required for such interaction. This unique structural characteristic of ANXA9 whose biological function is not regulated by calcium, subsequently, leads to distinct subcellular locations, biochemical properties and biological functions beyond the scaffold features shared by other family members (Morgan et al., 1999a; Gerke and Moss, 2002; Goebeler et al., 2003).

Little is known about ANXA9 biological functions. Of special interest for epithelial biology is the finding that ANXA9 interacts with the N-terminal domain of periplakin, a protein that is especially relevant in the *epidermis* for the formation of epidermal cornified envelopes, and as a scaffold for several cytoskeleton proteins (Boczonadi and Määttä, 2012).

ANXA9 dysfunctions have been reported in several human cancers, showing a protumor role mainly linked to invasion and metastasis processes. ANXA9 expression has been involved in colorectal cancer invasion and metastases by regulating genes such as ADAM17, MMP-9, TIMP-1 and E-cadherin (Yu et al., 2018), and proposed as an independent factor of poor prognosis (Miyoshi et al., 2014; Yu et al., 2018). Likewise, ANXA9 expression has been associated with a higher incidence of bone metastasis in breast cancer and so included in a predictive gene panel (Cosphiadi et al., 2018), and related to breast cancer prognosis (Xiao et al., 2019).

In gastric cancer, ANXA9 upregulation has also been associated to poor prognosis in gastric cancer patients. In addition, ANXA9 was able to promote cell migration and growth in gastric cancer cell lines via TGF-β signaling, which reinforces its oncogenic role in these tumors (Zhou et al., 2021). ANXA9 expression was also associated with a poor prognosis in ovarian cancer and resistance to cisplatin *in vitro* and *in vivo*, posing ANXA9 as an interesting candidate for targeted therapies to overcome cisplatin-resistant cancers (Kou et al., 2021). Given that cisplatin-based chemotherapy is commonly used to treat HNSCC patients, it would be of major interest to address the role and expression of ANXA9 in cisplatin resistance in these tumors.

In the context of HNC, our group was first to evaluate by immunohistochemistry ANXA9 protein expression using a large homogeneous cohort of 372 surgically treated HPV-negative HNSCC patients. In normal tissues, ANXA9 expression was observed in the most differentiated layers of the squamous stratified epithelium but not proliferative basal cells. ANXA9 expression was downregulated in 42% of HNSCC samples compared to normal epithelia. This result was also further confirmed using transcriptomic data from the TCGA, which suggests possible transcriptional mechanisms underlying ANXA9 downregulation in HNSCC (Salom et al., 2019). Noteworthy, positive ANXA9 expression HNSCC was tightly associated with the histological differentiation grade, predominantly detected in well-differentiated tumors and oropharyngeal tumor location (Salom et al., 2019).

## Annexin A10

Annexin A10 (ANXA10) was first identified in 1999 (Morgan et al., 1999b). Similar to ANXA9, changes and inactivation of well-conserved calcium binding sites provide unique membrane-binding properties and calcium-independent functions to ANXA9 and ANXA10, suggesting that both proteins could act in environments where calcium sensitivity is not functionally determining (Morgan et al., 1999b; Gerke and Moss, 2002). Some of these known singular functions for ANXA10 are related to a nuclear subcellular location, regulation of transcription and mRNA processing (Quiskamp et al., 2014).

ANXA10 plays a dual role in human cancers. Although most malignancies exhibit an oncopromoter role, there are also evidences for a tumor-suppressor role in several cancers, such as hepatocellular carcinoma, gastric cancer, bladder cancer and lung cancer. ANXA10 downregulation in hepatocellular carcinoma has been associated with poor survival rates, higher early intrahepatic tumor recurrence, higher vascular infiltration and a higher grade. Interestingly, this downregulation was correlated with mutated p53, and both alterations synergistically contributed to tumor progression (Liu et al., 2002, 2012). Loss of ANXA10 expression is frequent in early gastric cancer, and constitutes an independent biomarker of poor prognosis (Ishikawa et al., 2020). Kim et al. found that ANXA10 may display a tumor-suppressor role by reducing tumor growth and promoting apoptosis in gastric cancer cells (KIM et al., 2010). It has also been suggested that ANXA10 may constitute a predictive biomarker of chemotherapy response in gastric cancer (Ishikawa et al., 2022a). In bladder cancer, low ANXA10 expression was associated with a shorter progression-free survival and proposed as a poor prognosis biomarker in both early and advanced tumors (Munksgaard et al., 2011). In addition, ANXA10 has been proposed to be a tumor suppressor in NSCLC based on migration and invasion assays and *in vivo* studies conducted by Hung et al. (Hung et al., 2019). Opposing to this, high ANXA10 expression was detected in lung adenocarcinomas, and promoted the migration of A549 cells (Yumura et al., 2022).

In relation to its oncopromoter role, ANXA10 has been proposed as a prognostic biomarker and therapeutic target in many tumor types. Of special interest, high ANXA10 expression was reported as an independent poor prognostic biomarker in lung adenocarcinoma (Yumura et al., 2022), glioblastoma multiforme (Xu et al., 2021), serous epithelial ovarian cancer (Wang et al., 2019), pancreatic ductal adenocarcinoma (Ishikawa et al., 2022b), small bowel adenocarcinoma (Ishikawa et al., 2021) and papillary thyroid cancer (Liu X. et al., 2021). Interestingly, ANXA10 has also been proposed as a useful and highly specific biomarker for adenocarcinomas of the upper gastrointestinal tract and pancreatobiliary system, and also included in a panel of biomarkers to trace the origin of adenocarcinomas with unknown primary sites (Lu et al., 2013).

High ANXA10 expression has been correlated with depth of invasion and poor disease-free survival in ESCC patients. Moreover, ANXA10 promoted growth of ESCC cell lines via activation of Akt and ERK1/2 pathways (Kodaira et al., 2019). ANXA10 expression was also correlated with poor overall survival in colorectal cancer (Bae et al., 2015), and intrahepatic cholangiocarcinoma (Shao et al., 2022). In addition, ANXA10 expression was found upregulated in primary and metastatic melanoma and associated with tumor progression, observations reinforced by its promoting role of cell migration *in vitro* and metastases *in vivo* (Zhang X. et al., 2021). ANXA10 expression has been postulated as a useful biomarker for early detection of pancreatic ductal adenocarcinoma since it can be detected in precancerous lesions (Zhu et al., 2017).

In HNC, ANXA10 upregulation was detected in primary OSCC compared to normal tissue, which was more frequent in advanced cases and associated to tumor size. Mechanistically, it has been reported that ANXA10 promotes G1 cell cycle progression and cell proliferation enhancing ERK phosphorylation and ERK/MAPK pathway activation (Shimizu et al., 2012). These findings support a protumor role of ANXA10 in oral tumorigenesis by promoting proliferation and tumor growth. In accordance to these data, results from our group confirmed ANXA10 upregulation both at protein and mRNA level in HNSCC tissue samples compared to the normal counterpart (Salom et al., 2019). Thus, ANXA10 protein was detected in 64% of the HNSCC specimens but absent in normal epithelia. ANXA10 expression was significantly associated with differentiation grade, and higher in oropharyngeal tumors; however, no correlations with HNSCC patient survival were observed (Salom et al., 2019).

## Annexin A11

Annexin A11 (ANXA11) displays a long N-terminal domain (aprox. 200 aminoacids), which resembles that of ANXA7, with a 3D structure that differs from the typical amphipathic helix motifs present in N-terminal domains from other family members. Together with ANXA7 and ANXA13, these are the earliest vertebrate annexins, and are considered the ancestor of the remaining annexin members (Gerke and Moss, 2002).

This protein is tightly involved in Ca2+-regulated exocytic processes, interrupting midbody formation during cytokinesis (and hence cell cycle progression), sex differentiation throughout gonad development and apoptosis regulation. Of note, ANXA11 interaction with S100A6 protein (calcyclin) is especially relevant for the aforementioned biological functions (Wang et al., 2014). As many other annexins, ANXA11 is involved in membrane curvature mechanisms, similar to ANXA7, and both proteins share their unique ability to form mobile lens structures in the bilayer (Boye et al., 2018).

ANXA11 dysfunction has been associated with several human diseases such as autoimmune diseases and thrombolysis (Wang et al., 2014), and especially with amyotrophic lateral sclerosis (ALS) (Siddique and T, 2001; Smith et al., 2017). Regarding human cancers, ANXA11 has been related to drug resistance and metastasis in several human malignancies, as discussed below.

ANXA11 acts as a tumor-suppressor in hepatocarcinoma regulating apoptosis, invasion and lymph node metastases. ANXA11 downregulation led to reduced apoptosis and enhanced invasion and metastatic abilities, as well as enhanced chemoresistance to 5-FU in both *in vitro* and *in vivo* models (Liu et al., 2015; Liu et al., 2016 S.). In bladder cancer, ANXA11 expression levels were decreased in tumor tissue compared to the normal counterpart, and together with other annexins, serves to identify luminal-subtype bladder tumors (Wu et al., 2021). Moreover, high ANXA11 levels were associated with a higher overall survival in bladder cancer patients (Yao et al., 2022), further supporting an oncosuppressor role in this malignancy.

ANXA11 was found upregulated in gastric tumors compared to normal tissue, and increased ANXA11 levels were significantly associated with tumor size and infiltration, lymph node metastasis, TNM stage and vascular invasion, emerging as a bad prognosis factor and a potential therapeutic target for this cancer type. *In vitro* ANXA11 silencing reduced cell proliferation and invasion and migration capabilities, supporting a protumor role in gastric cancer (Hua et al., 2018).

ANXA11 has been involved in chemotherapy resistance in several malignancies. Downregulated ANXA11 expression is associated with cisplatin resistance in ovarian cancer, and pointed as a useful biomarker to predict chemoresistance to platinum-based therapies and early tumor recurrence (Song et al., 2007). Given that cisplatin is the gold-standard therapy for HNC patients, it would be interesting to explore the possible implication of ANXA11 in HNC chemoresistance, and its potential as a predictive biomarker of cisplatin response. Even though ANXA11 expression has not been detected in HNC tissue samples (Song et al., 2007), the small sample size in the only published study demands further investigation.

ANXA11 has also been associated with chemotherapy response in metastatic colorectal cancer, and ANXA11 nsSNP rs1049550 has been proposed as a biomarker to predict bevacizumab sensitivity in these patients (Kim et al., 2011, 2013; Roh et al., 2016). ANXA11 is overexpressed in colon primary tumors compared to normal colon, and associated with tumor stage. Moreover, patients harboring high ANXA11 and ANXA4 expression displayed poorer survival than those with low annexin expression (Duncan et al., 2008). High ANXA11 mRNA levels have also been associated to a poor overall survival in patients with acute myeloid leukemia, and related to a resistance mechanism to DR5-targeting agents that induce apoptosis regulation (Song et al., 2022). Since ANXA11 plays a role in apoptosis modulation, it could represent an important player in cancer therapies based on targeting apoptotic pathways and/or the development of resistance mechanisms, and hence emerge as a valuable biomarker to predict treatment response or to identify/stratify the subset of patients who are likely to respond (Song et al., 2022).

## Annexin A13

Annexin A13 (ANXA13) is the earliest annexin ancestor in vertebrates. Its expression is tightly restricted to intestine, and transcribed as two isoforms by alternative splicing (named A13a and A13b) (Iglesias et al., 2001; Gerke and Moss, 2002). Of note, it is the only member myristoylated at its N-terminal domain, allowing specific membrane association features that may act synergistically with canonical (Gerke and Moss, 2002; Turnay et al., 2005; McCulloch et al., 2019). Like other annexins, ANXA13 is involved in membrane biology, being of special relevance in lipid-raft dynamics involved in apical transport (Lafont et al., 1998; Plant et al., 2000).

In cancer, high ANXA13 expression has been associated with a poor outcome in colorectal cancer (Jiang et al., 2017), and lung adenocarcinoma where it promotes cell migration, invasion and EMT (Xue et al., 2020). To date, the clinical significance of ANXA13 in HNC remains unexplored.

## Concluding remarks

According to the herein reviewed data, dysregulation of annexin expression is a common feature in multiple cancers (Table 1 and Figure 2) that causes widespread functional effects on multiple cellular processes critical for tumor biology, thereby affecting major hallmarks of cancer (Table 2 and Figure 3). Hence, it is not surprising that this family of proteins offers enormous opportunities for the identification of clinically relevant biomarkers and novel molecular targets for the development of personalized therapies. In fact, this review compiles comprehensive information evidencing the great potential of annexins as biomarkers for cancer diagnosis, prognosis, disease monitoring, prediction of treatment response and/or therapeutic targets (summarized in Table 3). Nevertheless, given that each protein exhibits varying expression levels and phenotypic specificity depending on the tumor type, in-depth functional and mechanistic characterization of annexin dysregulation for each individual cancer poses a fundamental task to safety translate this knowledge into clinical application.

Annexins display different expression patterns, some of them are found upregulated in certain tumor types while downregulated in others, and such imbalance may trigger a variety of downstream signaling pathways and functional regulatory effects. In addition, annexins show important differences in their calcium binding ability, subcellular locations, post-translational modifications and a high number of interaction partners. All these factors jointly contribute to complicate their precise functions in each cell and tissue. Ultimately, annexins cannot be overall classified as an oncogene or a tumor suppressor, they should actually be considered as double-edged swords depending on the cellular or tissue context.

HNC encompasses a complex and heterogeneous group of aggressive malignancies, whose main pitfalls are late diagnosis, poor prognostication, scarce molecular-targeted therapies, and drug resistance. Therefore, annexin research represents a growing area of interest to attempt to overcome the major challenges of this disease. Based on the existing published data, ANXA1 and ANXA2 are the most extensively investigated, revealed as both clinically and biologically relevant and, as such, highly promising for clinical application. It has been demonstrated that these two proteins participate in numerous hallmarks of cancer (Figure 3), which highlights their central role as key players and possible targets for therapy in HNC as in other cancers.

Even though targeted therapies based on annexin dysfunctions are not yet available for cancer treatment, annexins are potentially druggable targets that could be therapeutically exploited. Precisely, various strategies have already been developed successfully targeting ANXA1 and ANXA2 functions, which have shown efficacy on various preclinical tumor models. It is therefore strongly recommended to further extend testing to HNC models. In particular, we may anticipate that ANXA2 blocking strategies might be adequate in NPC and OSCC to specifically target its oncopromoter role, but not in other HNSCC subtypes, such as laryngeal and pharyngeal carcinomas, where ANXA2 expression is downregulated and oncosuppressive. This should also be taken into account when eventually designing clinical trials in the future, in order to adequately define the inclusion criteria, to optimize patients’ stratification and enrollment, and ultimately to maximize clinical efficacy as well as patient benefit and safety. Similarly, the ANXA1 mimetic peptide Ac 2-26 (with reported cardioprotective effects) could also represent a valuable therapeutic option for some HNC subtypes; however, this possibility requires in-depth functional characterization in disease-relevant preclinical models.

It is worth highlighting that ANXA1 and ANXA2 have been linked to radio-chemoresistance and immunosuppression in HNC and other cancers, which poses a plausible application as adjuvant treatments to jointly improve treatment response and antitumor immune response. Nevertheless, the mechanisms responsible for these immunomodulatory actions need a more precise elucidation, since different results have been reported depending on the experimental settings and cellular models tested. Other family members (i.e., ANXA4, ANXA5, and ANXA7) have been associated to treatment resistance mechanisms in HNC, which also merits deeper investigation.

On the other hand, from a biomarker point of view, various annexins have been pointed as potential diagnostic, prognostic and/or predictive biomarkers in HNC. Of great interest and application, results revealing ANXA1 lost as an early marker to facilitate histopathological diagnosis of epithelial dysplasia (Garcia Pedrero et al., 2004). This molecular test based on immunohistochemical detection has shown a 100% sensitivity, and it could be easily implemented to routine clinical practice. ANXA1 (Garcia Pedrero et al., 2004), A2 (Rodrigo et al., 2011b, 2014) and A9 (Salom et al., 2019) expression has been closely related to histopathological grading in HNSCC. Interestingly, since ANXA1 can be externalized (Cooray et al., 2013; Gastardelo et al., 2014), its expression is detectable in liquid biopsy, changes upon treatment, and ANXA1 mRNA levels have been proposed as a blood-based biomarker for OSCC detection (Faria et al., 2010). In addition, ANXA8 mRNA expression levels have been postulated as a biomarker to detect OSCC lymph node metastases histopathologically undetectable (Oka et al., 2016).
